# The Role of Footwear in the Pathogenesis of Hallux Valgus: A Proof-of-Concept Finite Element Analysis in Recent Humans and *Homo naledi*

**DOI:** 10.3389/fbioe.2020.00648

**Published:** 2020-06-30

**Authors:** Genyu Yu, Yuzhou Fan, Yuxuan Fan, Ruining Li, Yaming Liu, Djordje Antonijevic, Petar Milovanovic, Bo Zhang, Zhiyu Li, Marija Djuric, Yifang Fan

**Affiliations:** ^1^Foot Research Laboratory, Key Laboratory of Sport and Health Science of Fujian Province, School of Physical Education and Sport Science, Fujian Normal University, Fuzhou, China; ^2^Shenzhen Tourism College, Jinan University, Shenzhen, China; ^3^Laboratory for Atomics Physics, Institute for Nuclear Sciences “Vinca”, University of Belgrade, Belgrade, Serbia; ^4^Laboratory for Anthropology, School of Medicine, Institute of Anatomy, University of Belgrade, Belgrade, Serbia; ^5^College of Foreign Studies, Jinan University, Guangzhou, China

**Keywords:** first metatarsophalangeal joint, hallux valgus, finite element, body coordinate, geometric model standardization

## Abstract

Hallux valgus (HV), the bunion of the first metatarsophalangeal joint (MTPJ), bothers many adults. No consensus has been reached about the causes of HV, be it a hereditary, or acquired, or multifactorial disease. Nor has agreement been reached using MTPJ angle to assess HV based on X-ray because in most cases the assessment of MTPJ is not reliable as it depends on the posture during scanning. In this study, we assume that HV is predominately acquired and that shoe wearing *per se* is an important player in HV pathogenesis. To verify our hypothesis, a CT-based finite element (FE) model of the first MTPJ of fossil remains of bear-footed *Homo naledi* was created and compared to that of five contemporary shoe-wearing wrestlers (10 models from two scans at an interval of about 18 months) because *Homo naledi*'s first MTPJ is an ideal model for non-shoe wearing with parallel sesamoid grooves. We developed the first MTPJ structure transformation method and created MTPJ joint capsule model for both *Homo naledi* and wrestlers. Constraint on the medial side of the first MTPJ capsule was set to simulate shoe-wearing conditions compared to the lack of medial constraint for barefooted conditions. Analysis of eight FE models of different angles for the first MTPJ of *Homo naledi* was performed by the first MTPJ transformation method and results showed that stress concentrated on the medial capsule of the first MTPJ in simulated shoe-wearing conditions, even at MTPJ angle of 0°. Increase in the first MTPJ angle further increased stress concentration on the medial side, and stress-growth relationship might reveal the causes of HV. We further developed a method to position the first MTPJ in wrestlers and created CT-based models at two time points. It was evident that the first MTPJ angle increased in all but one athlete, with a maximal increase of 4.03 degrees. This verifies our hypothesis that HV might be developed by wearing shoes. Further longitudinal studies with larger sample sizes are needed to additionally validate our results and determine the magnitude of the effects of shoe wearing on development and progression of HV.

## Introduction

Hallux valgus (HV), the bunion of the first metatarsophalangeal joint (MTPJ), is the most common foot deformity leading patients to visit a podiatric specialist in Europe (Crevoisier et al., 2016). Its prevalence increases with age (Nix et al., 2010; Dufour et al., 2014; González-Martín et al., 2017; Rodríguez-Sanz et al., 2018). Apart from pain and esthetic problems, HV might increase the risk of falling in the elderly (Menz et al., 2006; Rubenstein, 2006; Mickle et al., 2009; Muchna et al., 2018), which may lead to serious injuries or at least restrictions in mobility (Sattin et al., 1990; Bagalà et al., 2012). The HV deformity occurs more commonly in females (Perera et al., 2011). Despite the numerous studies the mechanism behind the HV remains controversial (Nix et al., 2010; Crevoisier et al., 2016), with agreement that the leading causes could be heredity (genetic) and environmental (ill-fitting shoe wearing) (Piqué-Vidal et al., 2007; Nguyen et al., 2010). The environmental contribution to HV pathogenesis (Lam and Hodgson, 1958; Kato and Watanabe, 1981) suggests that the deformity is preventable. However, the etiology of HV is mainly unknown. The association of HV with shoe wearing is not investigated in detail. This is important to consider in modern population having in mind that footwear plays an instrumental role in our daily life by providing us with safety and comfort, foot protection, prevention of slipping, and absorbing ambulatory shock, but wearing ill-fitting shoes was shown to lead to foot deformities (including HV) among 60% adults (Klein et al., 2009). Children without a habitual footwear developed better motor skills such as jumping than those wearing shoes (Zech et al., 2018). Indeed, the incidence of HV is very low in some non-shoe-wearing regions (Lam and Hodgson, 1958; Kura et al., 1998), indicating that shoe wearing might be the leading cause of HV. In addition, no fossil footprint was reported with HV. Taken together, these might suggest that ill-fitting shoe wearing is the leading cause in the pathogenesis of HV. However, HV can develop even in individuals who wear comfortable and fit shoes (Groarke et al., 2012), and therefore, here we hypothesized that even wearing appropriate footwear could contribute to the development of HV deformity.

When diagnosing HV via the X-ray, MTPJ angle has been considered a morphological indicator (Karasick and Wapner, 1990). Five methods were proposed regarding angle measurement and their accuracy and accountability remain controversial (Kura et al., 1998). Therefore, for higher accuracy, here we reconstructed the CT-based first MTPJ to measure the first MTPJ angle. The current study was designed to investigate how shoe wearing contributes to pathogenesis of HV. In general, biomechanical analyses are employed to clarify how external stress affects the morphology and structure of foot bones (Frost, 1994; Fung, 2013). When analyzing the mechanism of HV development, two critical steps should be taken. One is to create a geometric model of the first metatarsal bone, phalanges of the great toe and their joints. Another issue is to include the joint capsule in the finite element (FE) analysis of the first MTPJ, which was missing in previous studies (Yu et al., 2008). Therefore, to address the question whether shoe wearing contributes to the occurrence of HV in this study, we developed geometric and FE model of the first MTPJ in *Homo naledi* (Berger et al., 2015) [an extinct hominine species who had no experience of shoe wearing, with parallel sesamoid grooves (Fan et al., 2019)] and of the first MTPJ of contemporary wrestlers, and evaluated their biomechanical characteristics in simulated shoe-wearing and barefooted conditions (Sousa and Tavares, 2014). Longitudinal follow-up of the contemporary wrestlers' MTPJ was undertaken to follow the real-life effect of footwear on HV development.

## Materials and Methods

### Samples and CT Scanning

This study was conducted upon the approval from the Ethics Committee of Fujian Normal University. The test was conducted under the approved guidelines. All human participants provided fully informed consent to participate in this study by signing a written consent form.

Foot bones of *Homo naledi* were retrieved from https://www.morphosource.org/. According to Harcourt-Smith et al. (2015), the fossil was scanned using the Next Engine desktop scanner. Codes from the foot remains are: U.W. 101-1443 Metatarsal 1, U.W. 101-1551 Distal 414 hallucial phalanx and U.W. 101-1419 Proximal hallucial phalanx. *Homo naledi* was chosen as an extinct hominine species who had no experience of shoe wearing.

In addition, this study recruited five male wrestlers without injury or skeletal muscle disease. Their mean age was 20 ± 3 years, mean height 166 ± 6 cm, and mean weight 60 ± 1 kg. Their foot bones were scanned with 64-sliced CT twice, with an interval of about 18 months. Both scans were performed with the same multi-slice CT scanner (Philips/Brilliance 64). The scanner settings were the same for both examinations: approximately 120 kVp and 50 mA. Participants were asked to remain in the standard anatomical position. CT images were reconstructed by the scan condition of bone window, 0.9 mm slice thickness with 0.45 mm slice increment, 768 × 768 pixels, field of view ranging from 258 to 410 and number of slices from 423 to 548. CT DICOM format data were exported into *Mimics* software (Mimics Research 17.0 for X64; Materialize, Leuven, Belgium), which were used to create 3D geometric model of metatarsals.

When standardizing MTPJ coordinate system, we retrieved the angle between the first metatarsal's long axis and the proximal phalanx's long axis. See Supplementary File (SF) Part IV for the positioning procedure.

Simulation was performed as follows: in barefooted condition, no geometric constraint was applied to the medial side of the first MTPJ capsule while in shoe-wearing condition, constraint was applied (Sousa et al., 2012). To build the geometric model of MTPJ that has no experience of wearing shoes, we chose the MTPJ (Fan et al., 2019) and mirrored medial cuneiform (Li et al., 2019) of *Homo naledi* to build 8 geometric models. This is not a complete reverse reconstruction model, but eight FE models of different angles. Literature shows that <14° is a mild bunion, 14–20° is a moderate bunion, and more than 20° is a severe bunion (Robinson and Limbers, 2005), so we choose 0, 3, 6, 9, 12, 15, 18, and 21° to reconstruct the first MTPJ angle of *Homo naledi*, using the first MTPJ transformation method. See SF Part VIII for the detailed description of the process.

Ideally, the MTPJ models should be homogeneous, isotropic, and linear elastic. The main contact conditions are “Bonded” and “No Separation.” Each model has approximately 50,000 nodes and 30,000 elements (Ansys 14.0, Ansys Inc., Canonsburg, PA, USA). Material properties are included in Table 1. See SF Part IX (Figure S8) for details.

**Table 1:** Material properties assigned in the finite element model.

**Component**	**Young's modulus (MPa)**	**Poisson's ratio**	**Element size (mm)**	**Element type**
Foot bone^*^	7,300	0.3	5	tetrahedra
Cartilage^*^	1	0.4	1	tetrahedra
Joint capsule	300	0.4	5	tetrahedra
Ground^*^	30,000	0.3	5	brick

* *Properties of the FE model of foot bone, cartilage, and ground are based on the data from the literature (Zhang et al., 2005) while those of the joint capsule are modified from Antunes et al. (2008)*.

Constraint conditions for bare-footed and shoe-wearing were shown in the SF Part IX where constraint zone was highlighted.

### Positioning Method to Metatarsophalangeal Joint Posture

Rotating coordinate system is a method to conduct mechanical analysis (Rabi et al., 1954). When reconstructing body coordinate system of the first MTPJ via Mimics, it is sensitive to the subject's foot scanning posture, i.e., postures from different subjects differ and postures from the same subject but different scanning times differ as well. To position the MTPJ postures from different subjects and from different times is a prerequisite to standardize the loading as well as a condition to draw the joint capsule (refer to SF Part IV and V) because only when the first MTPJ's body coordinate system is standardized would the force loading between different subjects and between different time have the same direction to improve the accuracy of force loading. The following procedures were performed in order to accurately position MTPJ:

- Standardize the body coordinate of the first metatarsal, the great toe's proximal and distal phalanx, respectively (SF Part I, II, and III).
- Calculate MTPJ's principal axes of Euler (PAE). Namely, we took the first metatarsal's PAE as the body coordinate of the first MTPJ and set the origin of the coordinate on the centroid, thereby standardizing the body coordinate of the first MTPJ bones (SF Part I, II, and IV).
- Rate the standardized body coordinate of the first metatarsal along the long axis by percentage, thereby to standardize the first metatarsal, the great toe proximal and distal phalanx geometrically (Li et al., 2019) (SF Part I, II, and V).
- Calculate the angle between the first metatarsal and the three PAEs of the great toe proximal phalanx. These three angles are labeled as α *β γ*. See SF Part I, II, and VI for the process.
- Mark the proximal joint coordinate of the great toe proximal phalanx long axis and draw a point on the coordinate (SF Part VI).
- Position the point drawn on the sagittal plane and draw the curvature circle of the first metatarsal head contours on the plane (SF Part VII).
- Draw a point at the center of the curvature circle. Let the great toe proximal and distal phalanx rotate β about axis *x* to standardize the phalange relative to the metatarsal bone on the coronal plane (SF Part VII).

### First Metatarsal Joint Structure Transformation Method

In the course of screening participants, we found all the first MTPJ's grooves for sesamoid bones lack parallelism. The parallelism of sesamoid grooves means best ergonomics of windlass effect, it boosts the function of the flexor hallucis brevis as well as flexor hallucis longus muscles. Therefore, parallel first MTPJ grooves are important. Shoe-wearing stress might lead to structural change, i.e., unparalleled first MTPJ grooves (Fan et al., 2019).

We took the transformational model of the first MTPJ of *Homo naledi* as an ideal model of the HV development of shoe-wearing and bare-footed conditions. Since the accurate diagnosis of HV depends on the MTPJ angle (Karasick and Wapner, 1990), the pathological feature of HV is an increase of the MTPJ angle, i.e., the first MTPJ bunion (Thomas and Barrington, 2003). Our hypothesis is that HV is an acquired disease by wearing shoes, which means that in the same loading, as the MTPJ angle increases, the stress of the first MTPJ increases accordingly. To simulate the pathological process, we created 8 FE models of *Homo naledi* in shoe-wearing and bare-footed conditions.

- To standardize the body coordinate system of the repaired first metatarsal, first proximal and distal phalange (Fan et al., 2019) and the mirrored medial cuneiform, i.e., to calculate PAE for first metatarsal by using the PAE as its own body coordinate and setting the origin on its centroid. Rate the standardized body coordinate of the first metatarsal along the long axis by percentage, thus standardize the first metatarsal, the great toe proximal and distal phalanx geometrically. The PAEs and their calculation, see literature (Li et al., 2019) (SF Part I and II).
- Calculate the PAEs of the great toe proximal phalanx and work out the angle between the PAE of first metatarsal and that of the proximal phalanx (SF Part VI).
- Unite the proximal and distal phalanx through Boolean operations and standardize the union of phalanxes on sagittal plane with the angle calculated (SF Part VII).
- Adjust the angle between the first MTPJ angle to zero degree on the transverse plane. Rotate the MTPJs 0, 3, 6, 9, 12, 15, 18, and 21 degrees, respectively, along the vertical axis, and obtain 8 geometric models of first MTPJ (SF Part VIII).
- Do a Boolean operation to each model of the union of phalanx and the first MTPJ, forming the union of the first MTPJ. Magnify the union of the first MTPJ and cut the union to form the first MTPJ capsule and the first interdigital joint capsule (SF Part IX).
- Establish the joint capsule of the first metatarsal and the medial cuneiform bone in the same way (SF Part IX).
- Design lateral and medial sesamoid based on the morphology of MTPJ grooves. Establish geometric constraints between the first metatarsal base and the medial cuneiform bone to achieve the similar movement of MTPJ during model loading, completing the FE geometric model with different angles of first MTPJ (SF Part IX).

### Standardizing the Loading

Elements of force include the magnitude, direction and point of application. To do an FE analysis to bone, the usual loading method is surface-based, i.e., to select the joint surface, define the magnitude and direction of force (Zhang et al., 2005). Actually, force loading can be point-based. It is known that a joint cavity lies between the head of a metatarsal and the base of the proximal phalanx. The joint capsule is a closed compartment filled with synovial fluid (Mow et al., 1993). The magnitude of the force on the inner wall of the joint capsule in the closed fluid compartment is the same, and the direction is outward along the line connecting the center of the force point and the center of curvature of the secondary point. This study loads point-based force (matrix) to the articular surface of the medial cuneiform facing the navicular.

- Take the lowest point of cuneonavicular joint fossa of the medial cuneiform bone as centroid (SF Part IX).
- Load force on the articular surface contour in the sagittal plane cross section, and the values of force are 5 × 50 N (Chen et al., 2012), pointing to the circle center of joint's curvature circle (SF Part IX.) See c) for how 50 points are obtained.
- Rotate 36 degrees in turn to form a 50-point-matrix force whose magnitude, direction and position are determined (SF Part IX).
- For boundary conditions for shoe-wearing, see SF Part IX.

The first MTPJ angle is defined as taking the vertical axis and the long axis of the first metatarsal as the sagittal plane of MTPJ. The angle between the long axis of the first metatarsal and the first proximal phalanx on the sagittal plane is the angle of MTPJ.

## Results and Discussion

**Figure 1:**
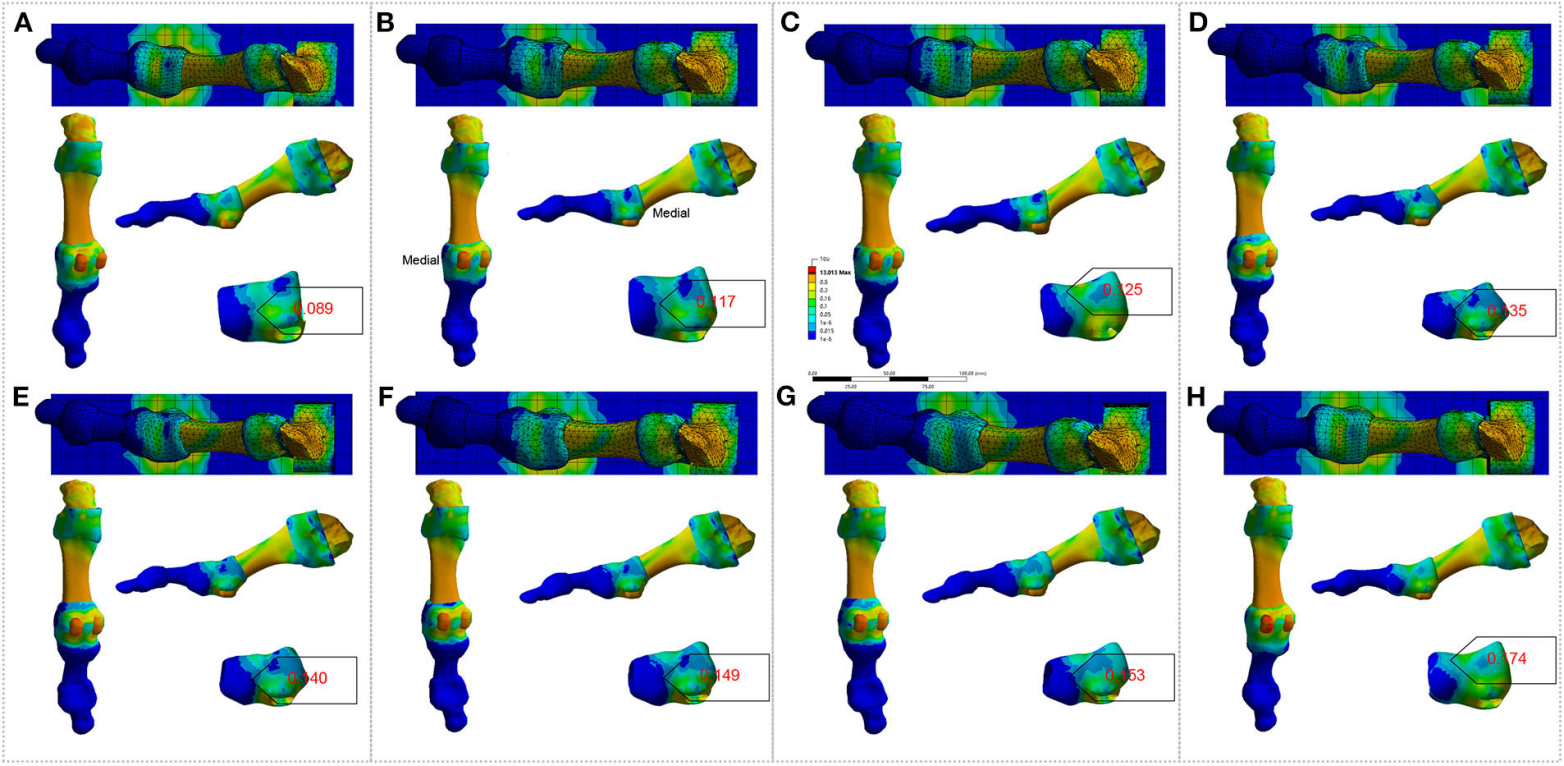
FE analysis to the right-sided first MTPJ of *Homo naledi* in bare-footed condition. **(A–H)**. FE analysis to MTPJ, with an MTPJ angle of 0, 3, 6, 9, 12, 15, 18 and 21 degree, respectively. Numbers in red refer to maximum values for the von Mises stress (unit: MPa).

**Figure 2:**
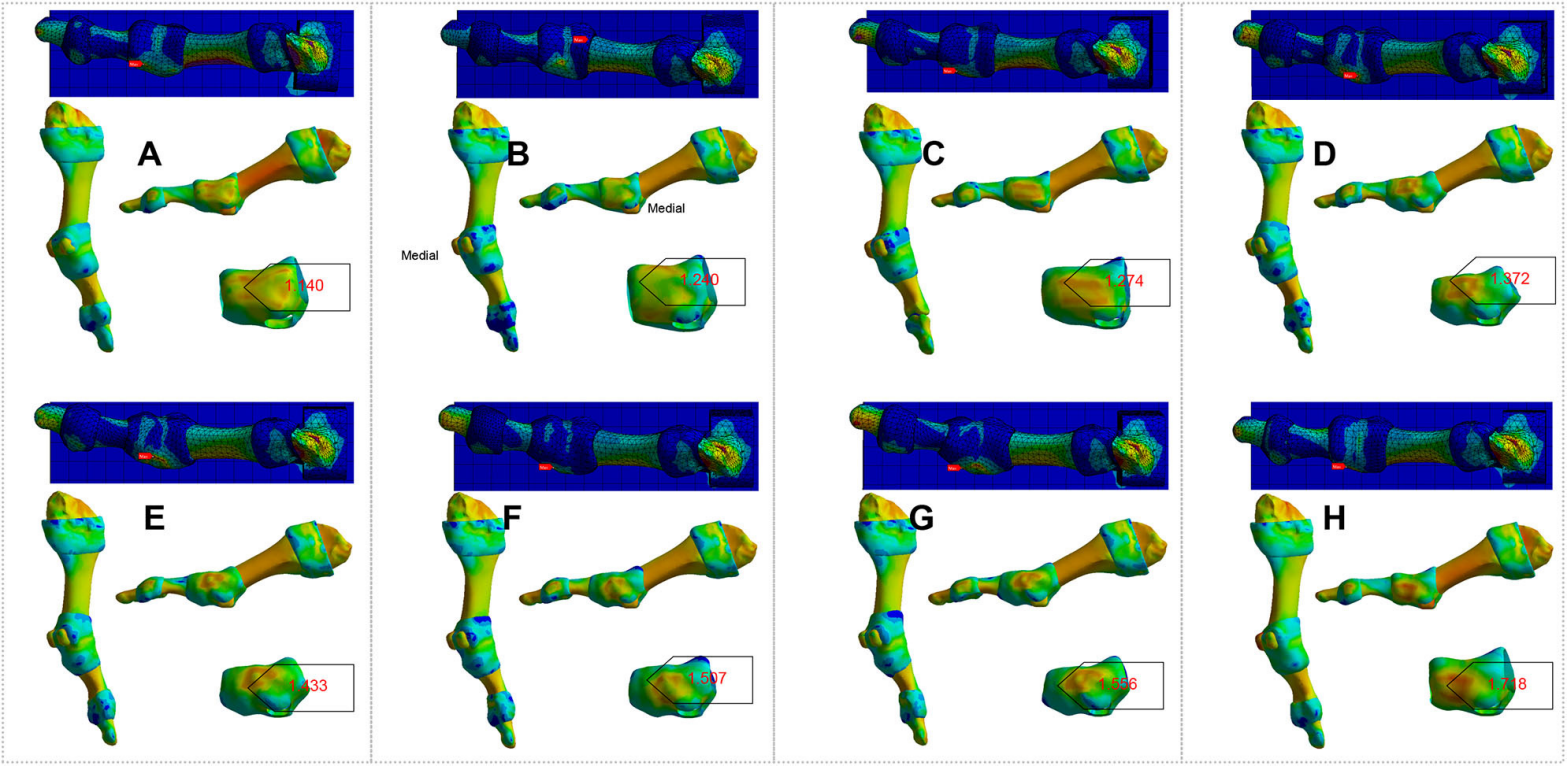
FE analysis to the right-sided first MTPJ of *Homo naledi* in shoe-wearing condition. **(A–H)**. FE analysis to MTPJ, with an MTPJ angle of 0, 3, 6, 9, 12, 15, 18, and 21 degree, respectively. Numbers in red refer to maximum values for the von Mises stress (unit: MPa).

FE analysis in *Homo naledi* revealed that as the first MTPJ's angle increases, the concentration of stress increases on the medial side of the first MTPJ's capsule both in barefooted (Figure 1) and simulated shoe-wearing conditions (Figure 2). However, it should be noted that the stress peak is much smaller in barefooted than in simulated shoe-wearing conditions (Figure 1 vs. Figure 2, Table 2). Namely, as shown in Table 2, the maximum and minimum principal stress of stress concentration in the medial aspect of the MTPJ capsule difference between simulated shoe-wearing and barefoot is several times while the Von Mises stress difference is about 10 times. It is obviously shown that when wearing shoes, comparing the angle of 21 and zero, the maximum, minimum and Von Mises stress difference is 2.26, 2.55, and 1.51 times, suggesting that as the HV angle increases, the maximum and minimum principal stress increase at the angle of 21. This suggests that exercise with shoes would directly increase the risk of HV development/progression. Specifically, wearing shoes sets geometric constraint onto the medial side of MTPJ and thus increased stress peak concentrates there during activities. Besides, different shoes' uppers with different elastic moduli might result in stress-shielding, which is likely to cause pathological changes to the first MTPJ capsule according to the stress-growth relationship (Fung, 2013) and lead to HV development and/or progression. In addition, SF Part XI shows that the relation between the first MTPJ angle and the stresses is linear. All of the coefficients are more than 0.9.

**Table 2:** Von Mises stress (VM), Maximum Principal Stress (Max-PS) and Minimum Principal Stress (Min-PS) of *Homo naledi*'s first MTPJ, simulated shoe-wearing (SW) & bare-footed (BF) conditions (unit: MPa).

**Boundary condition**	**Stress type**	**HV angle**							
		**0^**°**^**	**3^**°**^**	**6^**°**^**	**9^**°**^**	**12^**°**^**	**15^**°**^**	**18^**°**^**	**21^**°**^**
SW	Max-PS	0.100	0.111	0.138	0.150	0.161	0.180	0.210	0.226
	Min-PS	−0.148	−0.164	−0.182	−0.201	−0.244	−0.262	−0.332	−0.377
	VM	1.140	1.240	1.274	1.372	1.433	1.507	1.556	1.718
BF	Max-PS	0.040	0.045	0.056	0.062	0.067	0.074	0.089	0.098
	Min-PS	−0.042	−0.047	−0.060	−0.066	−0.074	−0.079	−0.100	−0.112
	VM	0.089	0.117	0.125	0.135	0.140	0.149	0.153	0.174

**Figure 3:**
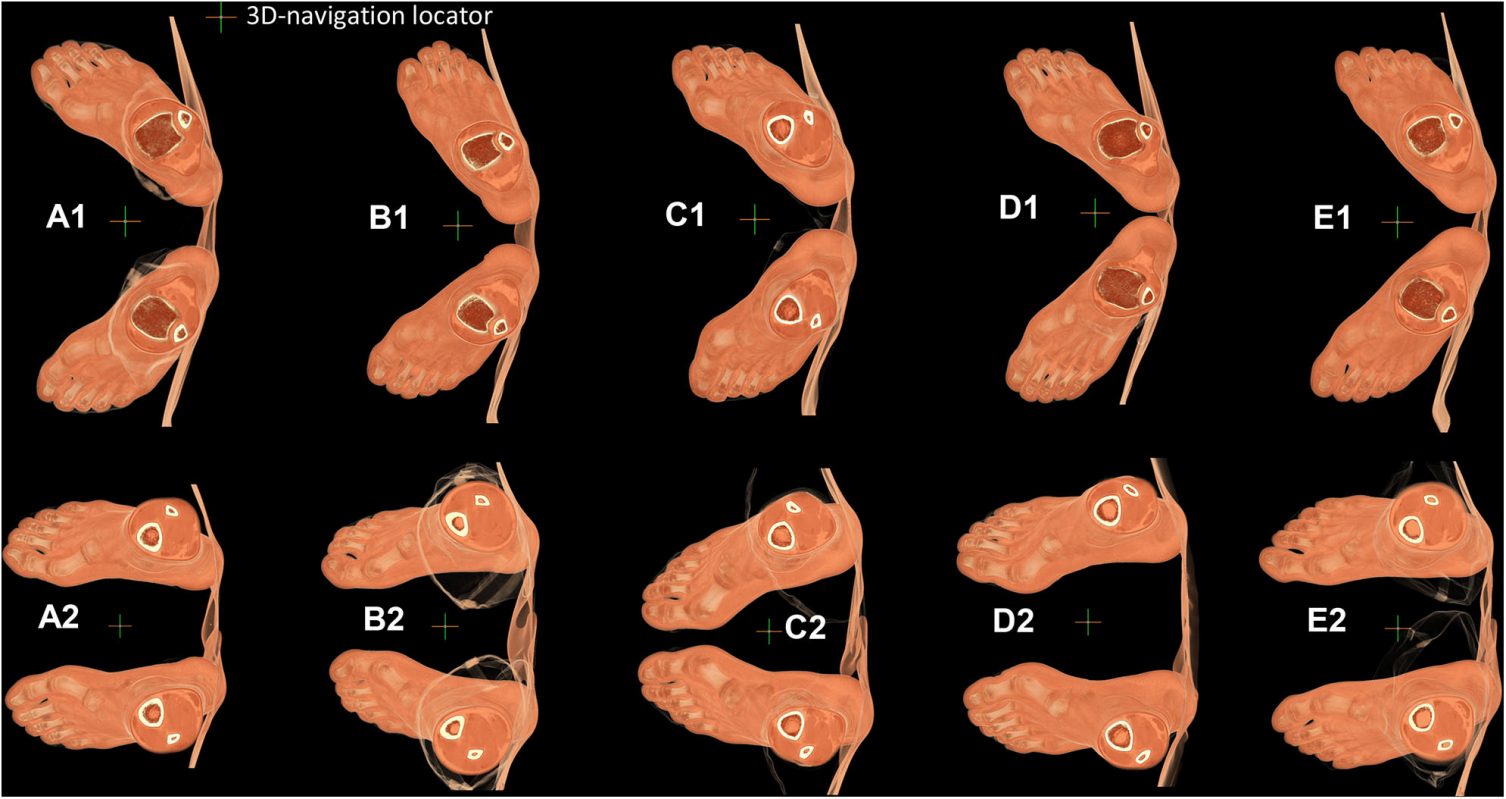
Each participant's posture of both feet obtained by 64-slice CT scanner. **(A–E)**. Feet of P1, P2, P3, P4, P5, respectively. **(A1–E1)**. Foot posture from the first time scan. **(A2–E2)**. Foot posture from the second time scan.

To verify our hypothesis, we did a longitudinal follow-up study on 12 on-training wrestlers. After an exclusion of those with injuries and retires, we got two times' scanning from 5 participants (at an interval of about 18 months): two individuals seemingly suffered HV while the remaining three had normal foot. See Figure 3 (participants labeled as P1 to P5).

**Figure 4:**
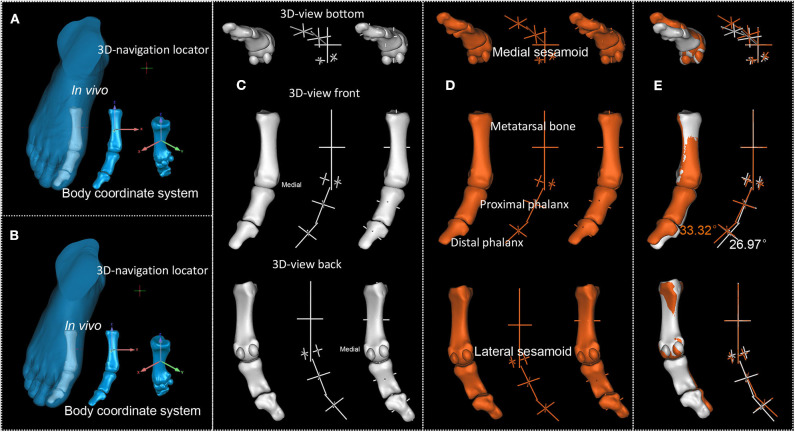
P1's first MTPJ. **(A,B)**. First and second scan result of P1's first MTPJ. **(C,D)**. First and second scan's reconstructed first MTPJ and the sphere-truss structure. **(E)**. Degree between the long axis of proximal phalanx of first MTPJ and the long axis of first metatarsal, white from the first time scan, orange from the second.

**Figure 5:**
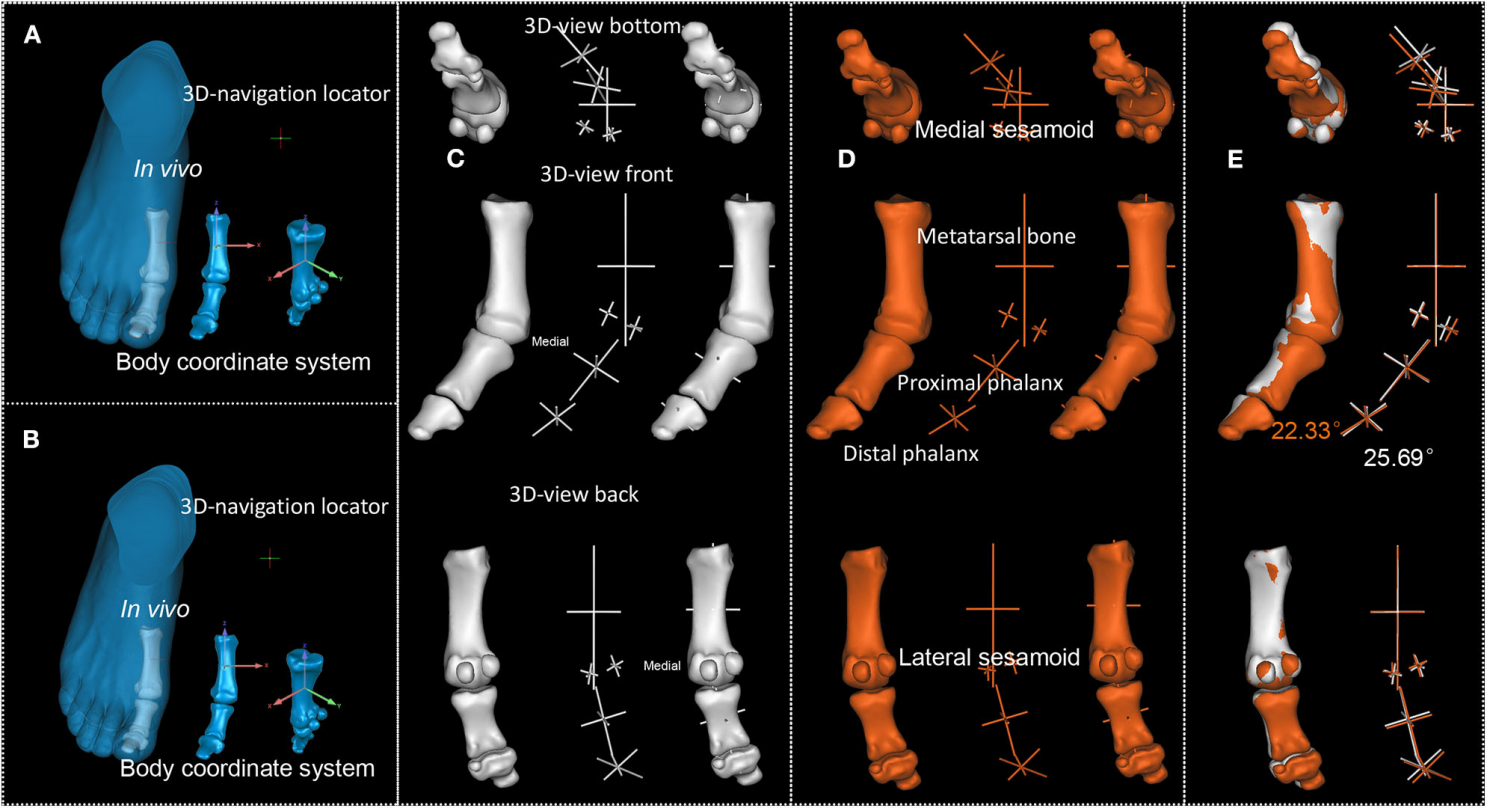
P2's first MTPJ. **(A,B)**. First and second scan result of P2's first MTPJ. **(C,D)**. First and second scan's reconstructed first MTPJ and the sphere-truss structure. **(E)**. Degree between the long axis of proximal phalanx of first MTPJ and the long axis of first metatarsal, white from the first time scan, orange from the second.

**Figure 6:**
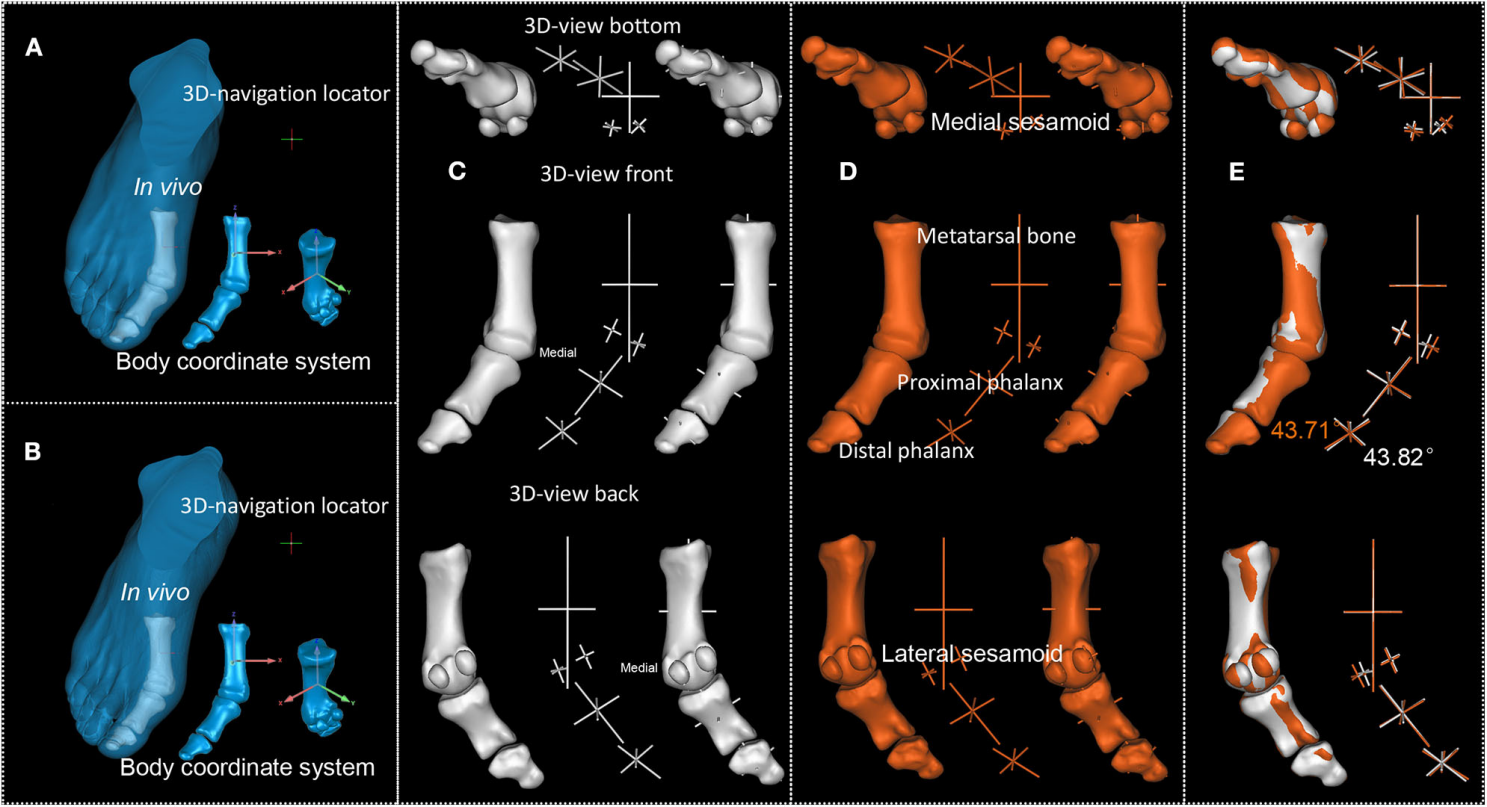
P3's first MTPJ. **(A,B)**. First and second scan result of P3's first MTPJ. **(C,D)**. First and second scan's reconstructed first MTPJ and the sphere-truss structure. **(E)**. Degree between the long axis of proximal phalanx of first MTPJ and the long axis of first metatarsal, white from the first time scan, orange from the second.

**Figure 7:**
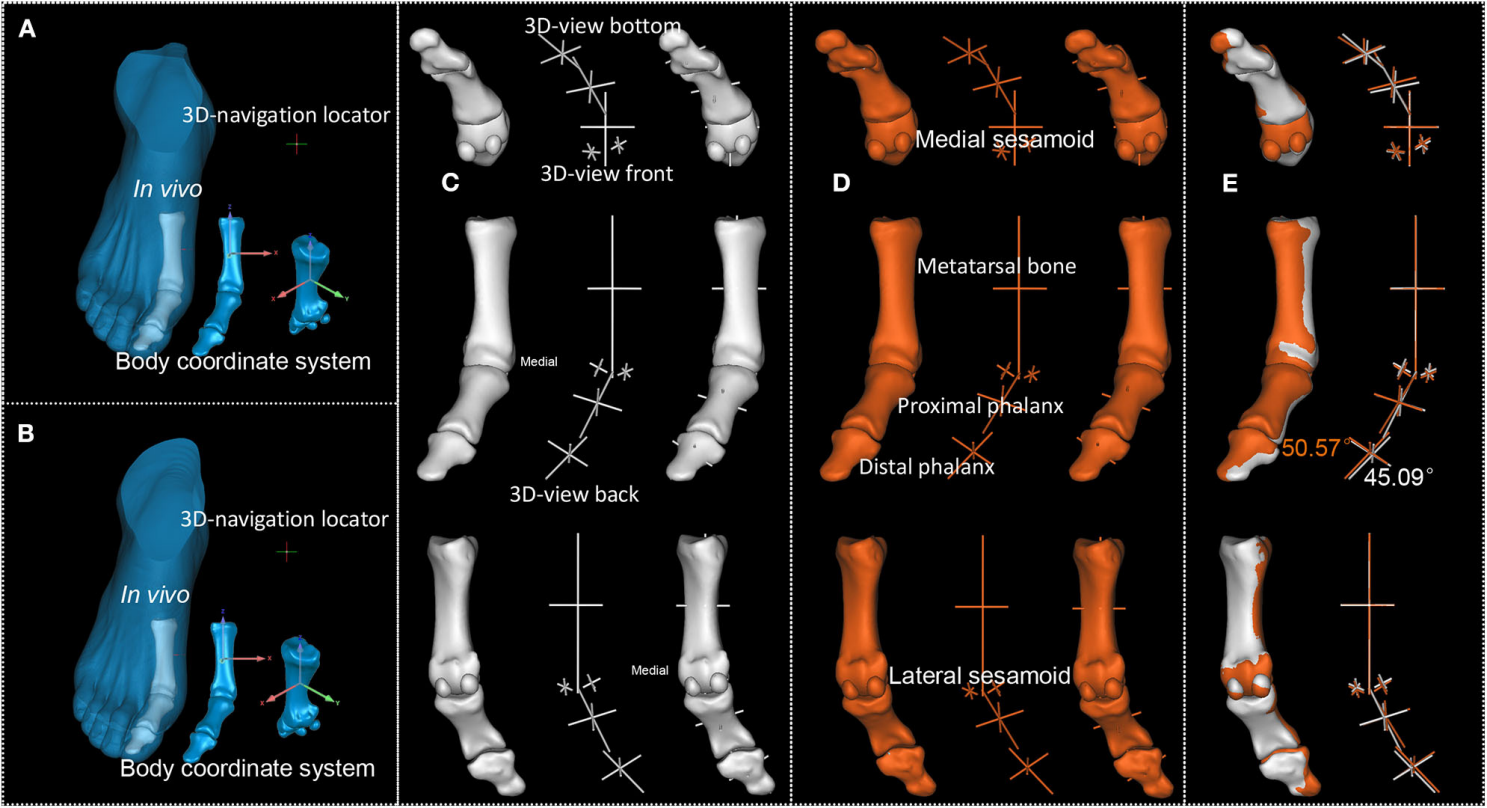
P4's first MTPJ. **(A,B)**. First and second scan result of P4's first MTPJ. **(C,D)**. First and second scan's reconstructed first MTPJ and the sphere-truss structure. **(E)** Degree between the long axis of proximal phalanx of first MTPJ and the long axis of first metatarsal, white for the first scan, orange for the second.

**Figure 8:**
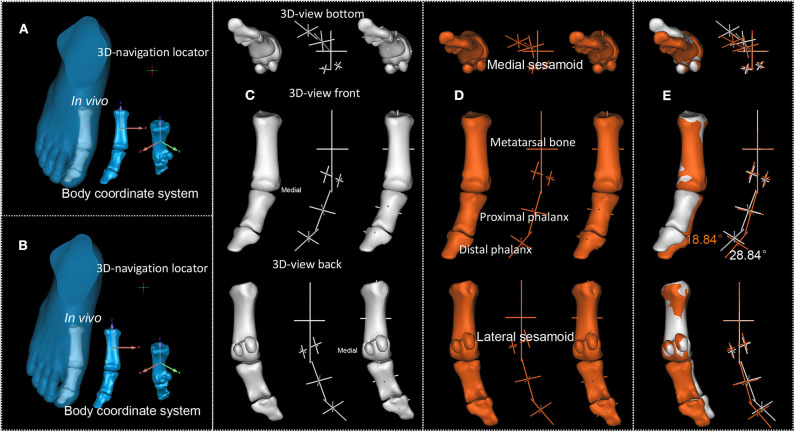
P5's first MTPJ. **(A,B)**. First and second scan result of P5's first MTPJ. **(C,D)**. First and second scan's reconstructed first MTPJ and the sphere-truss structure. **(E)**. Degree between the long axis of proximal phalanx of first MTPJ and the long axis of first metatarsal, white from the first time scan, orange from the second.

Comparison between Figures 3A1–E1 and Figures 3A2–E2 shows that the first MTPJ angle drawn from X-ray could be affected by the scanning posture (Kura et al., 1998). So, we need standardization. To standardize loading on the medial cuneiform, we standardize the body coordinate system as described in Material and methods. Figures 4–8 present the results of standardization of body coordinate system and sphere-truss structure of two scans. Sphere-truss structure enables us to get the angle between two straight lines. See SF Part VI Eq (58) for the calculating formula and SF Part VI presents the calculating process.

It can be observed from Figures 4–8 that the first MTPJ angle of P3 did not change between the two scans, while that of P4 increased even 5 degrees in the second scan. Both P3 and P4 seemingly had valgus MTPJ. The first MTPJ angle increased by 5.35 degrees in one of the three bunions but decreased in the other two. During the interval of about 18 months, wrestlers continued participating in training and competition, and none of them received any HV therapy. Based on the principle that the structure follows its function, we did FE analysis to the first MTPJ of five participants to verify the validity of the observed first MTPJ angle.

The ethics guidelines restrict the healthy people from scanning more than once a year. That explains why we could not ask the participants to be scanned both with and without shoes. In addition, even when wearing shoes, we cannot ensure that different participants have the same force loaded on their first MTPJ. Therefore, all participants were scanned barefoot, and we simulated shoe-wearing and non-shoe-wearing by loading geometric constraints or without loading. Specifically, in mechanical terms, shoe-wearing and bare-footed conditions correspond to having geometric constrains or not, respectively.

The first MTPJ angles in three axes (*x, y*, and *z*) of five wrestlers at two time points are shown in Table 3. Considering that shifts of angle can happen in all three axes, determining single MTPJ angle is not optimal. The first MTPJ angle (i.e., the angle between the long axis of the first metatarsal and the proximal phalanx) of five wrestlers changed after about 18 months, i.e., 6.35,−3.36,−0.11, 5.48,−10, respectively.

**Table 3:** Right-side first MTPJ angle from each participant (unit: degree).

**Item**		**P1**		**P2**		**P3**		**P4**		**P5**	
		**1st**	**2nd**	**1st**	**2nd**	**1st**	**2nd**	**1st**	**2nd**	**1st**	**2nd**
Metatarsal bone & proximal phalanx	x-axis	20.49	25.05	20.17	18.17	35.01	36.77	20.75	23.23	19.64	20.06
	y-axis	24.36	27.45	24.36	18.35	40.12	31.36	40.12	45.07	24.36	18.91
	z-axis	26.97	33.32	25.69	22.33	43.82	43.71	45.09	50.57	28.84	18.84

**Figure 9:**
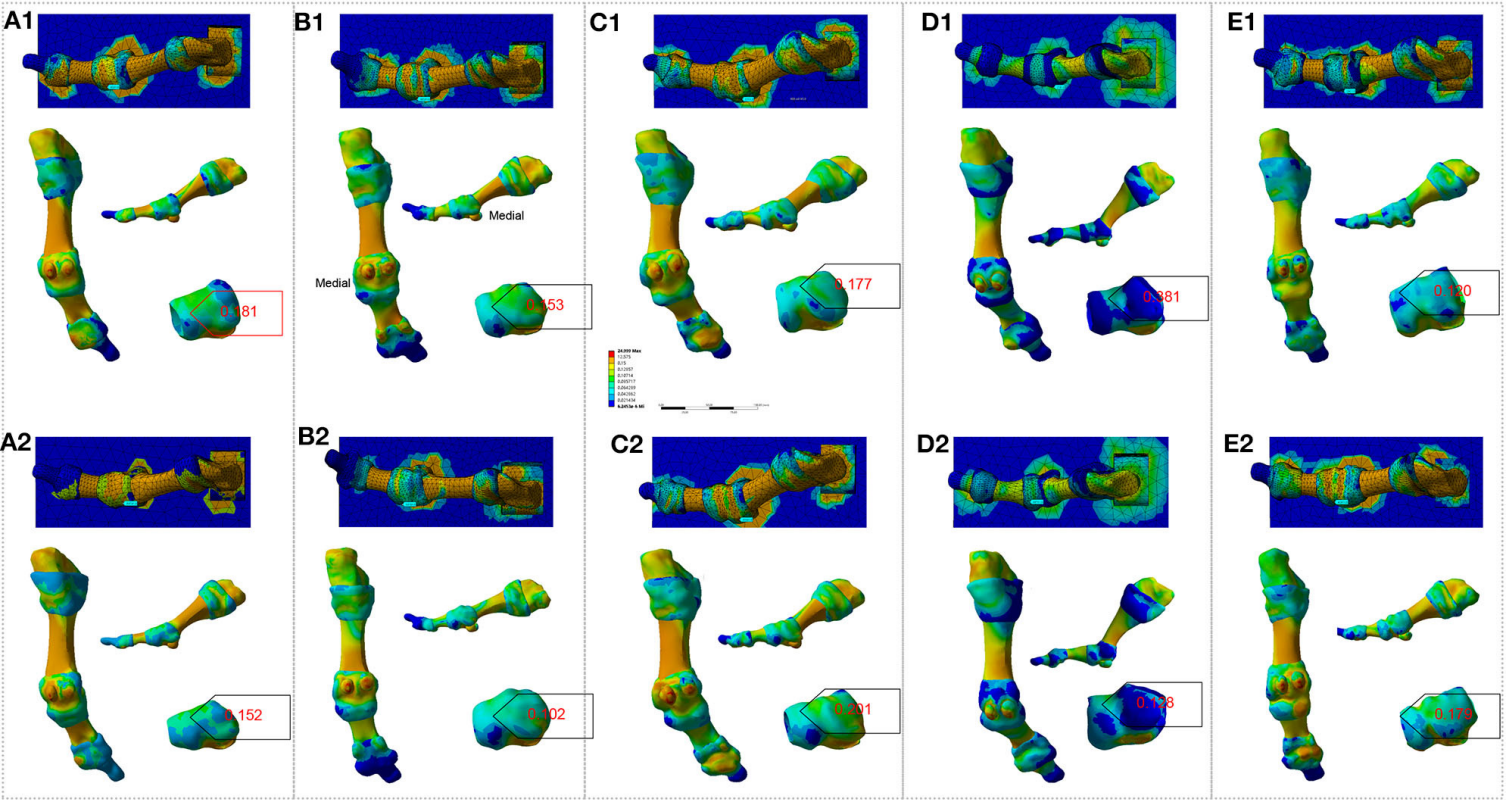
FE analysis to the participant's right-side first MTPJ in bare-footed condition. **(A1–E1)**. Analysis result of P1 – P5, respectively, from their first scan. **(A2–E2)**. Analysis result of P1 – P5, respectively, from their second scan. Numbers in red refer to maximum values for the Von Mises stress (unit: MPa).

**Figure 10:**
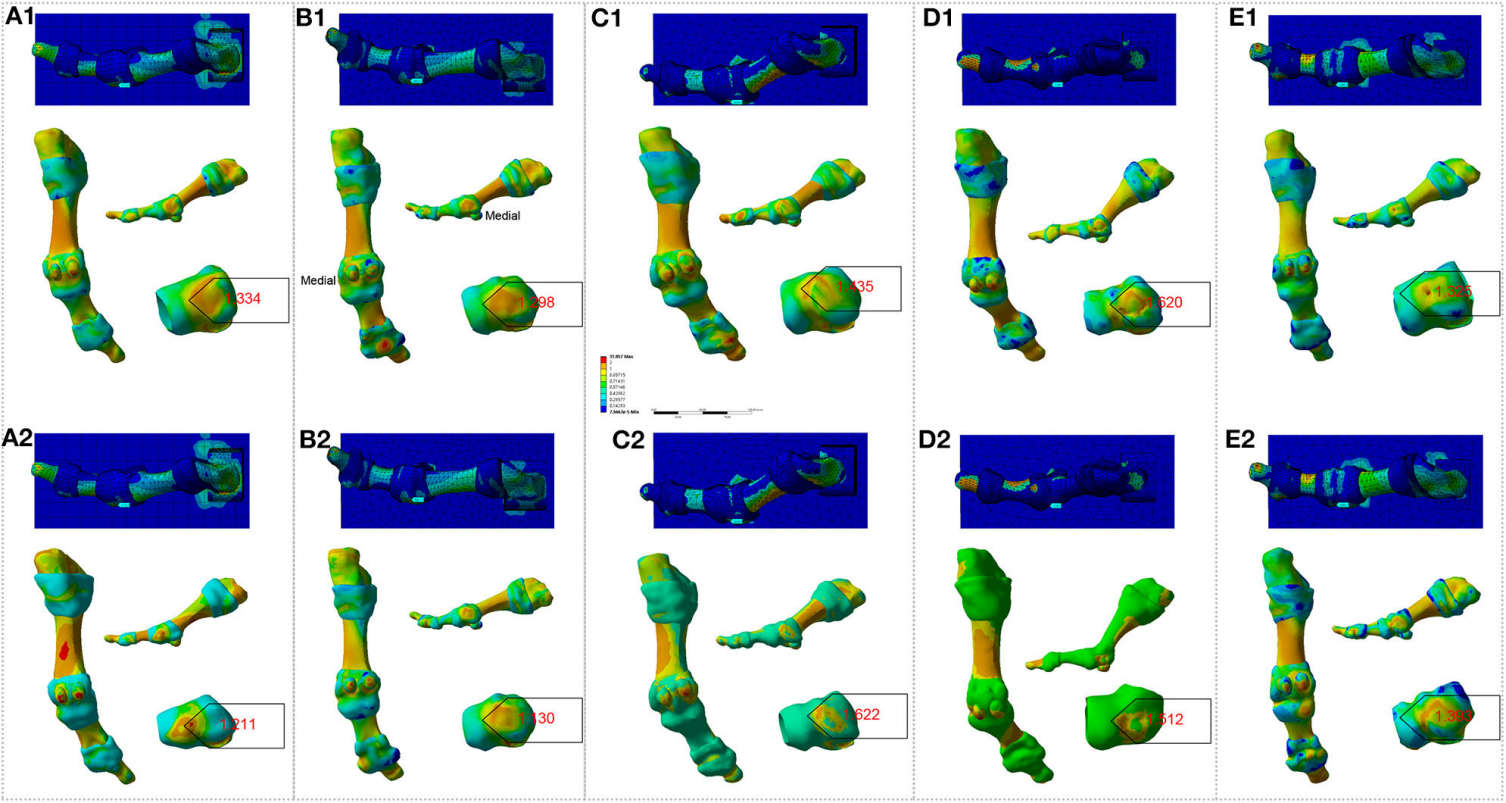
FE analysis to the participant's right-side first MTPJ in shoe-wearing condition. **(A1–E1)**. Analysis results of P1–P5, respectively, from their first scan. **(A2–E2)**. Analysis results of P1–P5, respectively, from their second scan. Numbers in red refer to maximum values for the Von Mises stress (unit: MPa).

To increase the accuracy, on the basis of body coordinate system of the first MTPJ, we standardized the geometric model and loading of FE analysis. The results of the analysis of being bare-footed and shoe-wearing conditions are shown in Figures 9, 10, respectively.

It is evident that there was some inconsistency in between Figures 4–8 and Figures 9, 10.

Figures 9, 10 show that the peak stresses on the medial capsule of the first MTPJ in the bare-footed and shoe-wearing condition are not in the same order of magnitude. The von Mises stress, maximum principal stress and minimum principal stress shows consistency in the same participant— i.e., the bare-footed and shoe-wearing of the same person are positively correlated. (See Table 2 and SF Part XI, Figure S10 for detailed information.) Shoes brought geometric constraints to the medial side of the first MTPJ. By loading matrix point-based force to the articular surface of the medial cuneiform facing the navicular, we simulated human locomotion. FE analysis presents difference between constrained and constraints-free conditions. Stress-growth relation reveals that shoe wearing increased stress on the medial side of the first MTPJ, resulting in over-growth of the medial side of the first MTPJ. Over-growth not only brings bunion to the first MTPJ capsule, but also presses the proximal phalanx to go outward, indicating that our FE model boundary conditions and loading model are feasible. Notably, it is difficult to correlate the first MTPJ angles in Figures 4, 8 and the results of the FE analysis in Figures 9, 10. Specifically, the joint angles of P1 and P4 increased, but no increase in peak stress on the medial side of capsule was observed. The MTPJ angle of P2 decreased, so did the peak stress on his MTPJ capsule. However, while also showing MTPJ angle reduction, in terms of stress concentration, P5 showed exactly the opposite to P2. The angle change of P3 was not obvious, but the stress peak increased significantly. This gave rise to the question whether the first MTPJ angle and FE analysis are suitable as criteria for evaluating hallux valgus.

**Figure 11:**
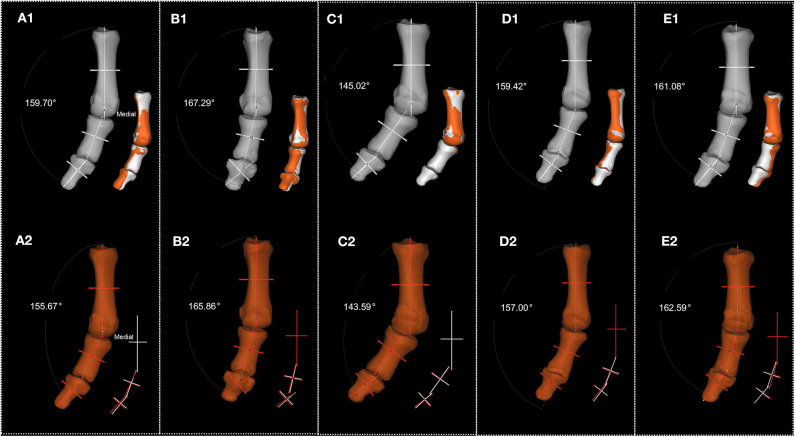
Standardization of each participant's phalanx posture. **(A1–E1)**. Standardization of each participant's reconstructed phalanx posture from their first scan (P1–P5, respectively). **(A2–E2)**. Standardization of each participant's reconstructed phalanx posture from their second scan (P1–P5, respectively). Numbers in white present the degree between the long axis of proximal phalanx of first MTPJ and the long axis of first metatarsal.

To clarify this question, we standardized the position of the proximal phalanx relative to the first metatarsal bone in coronal plane. Figure 11 and Table 4 show the degree between the first metatarsal and the first proximal phalanx in the coronal plane after standardization, demonstrating that except P5 whose joint angle reduced 1.15 degree, the first MTPJ angle of the all other participants increased after about 18 months, i.e., 4.03, 1.43, 1.43, 2.42, and−1.51, respectively. Tables 3, 4 show that the magnitude of the first MTPJ angle is influenced by the scanning posture. To eliminate such influence will make the variation of the first MTPJ angle more accountable. In Table 3, three wrestlers' first MTPJ angles all decreased, suggesting that training with shoe-wearing can treat HV. This is not realistic, nor theoretically supported. P5's reduction of the first MTPJ angle cannot be justified, and he had a suspension of training for a couple of months due to an avulsion fracture (See SF Part X Figure S9). Having noticed the uniqueness of P5, we observed his first MTPJ carefully and found osteophytes in the medial aspect of his proximal phalanx, where extensor hallucis longus was attached. When asked about this osteophyte, P5 attributed it to an injury that had happened during a competition about 10 months ago. The injury prevented him from training for a while and he had to wear simple shoes such as flip-flops in off-training period. This suggests that undertaking professional sport training in shoes increases the first MTPJ angle and could further develop to HV.

**Table 4:** Right-side first MTPJ positioned angle from each participant (unit: degree)^*^.

**Item**		**P1**		**P2**		**P3**		**P4**		**P5**	
		**1st**	**2nd**	**1st**	**2nd**	**1st**	**2nd**	**1st**	**2nd**	**1st**	**2nd**
Metatarsal bone & proximal phalanx	x-axis	20.49	25.05	20.17	18.17	35.01	36.77	20.75	23.23	19.64	20.06
	y-axis	2.89	6.60	16.37	11.81	1.52	5.95	2.68	3.31	5.62	13.22
	z-axis	20.30	24.33	12.71	14.14	34.98	36.41	20.58	23.00	18.92	17.41

* *The rotation is about axis x (See SF Part VIII), so in Tables 3, 4, the value of axis x (P1–P5) is the same*.

The question arises whether the high prevalence of HV (Nix et al., 2010) is due to the structural reason. It is known that the medial side of the first MTPJ capsule has abductor hallucis, with medial metatarsosesamoid ligament and metatarsosesamoid collateral ligament running across. In addition, the deep transverse metatarsal ligaments can anchor the relationship between the first MTPJ and the other MTPJs, with its attachment on the first metatarsal head (Stainsby, 1997), while the flexor tendon sheath (annular and cruciform parts) can barely function as a stabilizer (Yao et al., 1994). These structures indicate that the MTPJ lacks an anti-HV structure. Is this a slip from evolution?

The answer is “no.” Fossil records provided one of the most direct and reliable morphological evidence for living things (Tuttle, 2019). As early as 550 million years ago, creatures changed their directions when necessary, so did the Homo erectus. Footprints are the results from the interaction between the foot and the surface. Footprint fossil indicated no signs of HV borne by australopithecine from 3.6 million years ago (Leakey et al., 1976; Raichlen et al., 2010) or early hominin from 1.5 million years ago (Bennett et al., 2009). Furthermore, evidence showed the evolving process where the gap between the first metatarsal and the second metatarsal from Siamang (Fleagle, 1976), gorillas (Day and Napier, 1965) and chimpanzees (Wunderlich and Ischinger, 2017) gradually narrowed. The foot structure of OH_8_ (Susman and Stern, 1982) and *Homo naledi* indicated the completion of foot arch evolution. Their forefoot was fan-shaped (Li et al., 2019), which was consistent with the footprint shape (Bennett et al., 2009). The fan-shaped forefoot increased the stability of their gait support phase, and it also decreased the peak value of stress to avoid motion injury (Leardini et al., 2000).

Observation to footprint fossil revealed that the early hominin's big toe was straight (Bennett et al., 2009), which was consistent with that of the habitually bare-footed modern people (Holowka et al., 2019) and that of the clog-wearing ancient Japanese people (Kato and Watanabe, 1981). Hence, walking and jogging bare-footed may be optimal because big toe works the best without shoe restrains; the risk of getting HV is minimal because the support surface has no contact with the first metatarsal bone's medial joint capsule. The forefoot has pad and plantar fascia, and it also has two sesamoid bones to bear the reaction force of the support phase, and the sesamoid coupled with the first metatarsal, which will not affect the joint capsule and no bunion will occur.

From about 45,000 years ago, people began to wear shoes (Trinkaus and Shang, 2008). The earliest known straw sandals dated back to 10,000 years before while the existing record of shoes dated back to 5,500 years ago (Pinhasi et al., 2010), which were similar to the north American moccasins (Bramble and Lieberman, 2004). Until 1970's, the running shoes became popular globally. Though technology has boomed, it did not reduce the incidence of foot and ankle diseases (Lieberman et al., 2010). Research results from the habitually shoe wearing and the habitually bare-footed (Lieberman et al., 2010) suggested that HV could be a malady of the rich (Campbell, 2004) and the question arises whether HV is caused by wearing the shoes.

The design of shoes includes the comfort and packaging of foot. The packaging of foot (Nigg et al., 2015) brings the largest stress to the medial side of the first MTPJ capsule, as shown in Figures 1, 2. Why would the concentrated stress from the medial side of the first MTPJ capsule be a risk factor for HV? Because in activities such as running and jumping, the windlass mechanism of the MTPJ (Hicks, 1954; Bolgla and Malone, 2004) plays a fundamental role. When wearing shoes, the elastic moduli of the shoe's uppers, the skin, the muscle tendons and ligaments are not consistent, resulting in stress-shielding (Wearing et al., 2006), and then increasing stress concentration on muscle tendons and ligaments (Sousa et al., 2014, 2016). In addition, when the MTPJ involves windlass, the shoes bring stress-shielding to the medial side of the first MTPJ other than bearing the pressure. When exercising, e.g., jogging, the repetitive stress concentration, stress-shielding, and stress shearing will lead to the MTPJ bunion because the stress-growth relationship proves it to be so. This is the same to the physiological phenomenon of compensation in lung, kidney and artery (Fung, 2013).

Meanwhile, the positions of the positive maximum principal stress and negative minimum principal stress on the joint capsule of simulated shoe wearing from Table 2 have been observed. See SF Part XII, Figure S11 for details. Table 2 and Figure S11 address this problem, i.e., when the medial capsule of the first MTPJ is geometrically constrained (e.g., shoe wearing) and when the medial cuneiform is subjected to a load (e.g., load caused by movement), the medial capsule of the first MTPJ receives a positive axial principal stress, and a negative axial principal stress near the maximum axial principal stress (also on the medial capsule of the first MTPJ), and the absolute values of these two forces are very close. The two largest and smallest positive and negative axial forces form a moment of force, which “tears” the medial side of the first MTPJ with the human body's periodic movement (e.g., walking and running), enlightening how HV can be induced by shoe wearing and exercise. This is the biomechanical explanation to the incidence of HV. It offers evidence that running bare-footed can raise plantar tactile sensitivity and reduce the impact force to the knees (Holowka et al., 2019), and it can prevent and treat HV.

X-ray simplifies the 3D structure of foot into static 2D pictures, and a series of evaluation methods of HV are based on 2D pictures. The reconstruction of CT scanning could visualize and help analyze the 3D structure of foot. New morphological analysis method will bring new discovery (Tamura et al., 2011). Our research, based on the principle of form following its function, considers joint motion as transformation of body coordinate within bones and conducted 3D dynamic analysis through reconstruction CT scanning. However, how the rotation with multiple axes transforms could be further investigated.

This study is limited in sample size, type of sports event and age range. Future study should investigate a variety of sports event participants with a wider age range. And it should explore how age difference might affect the development of HV. Also, bone *in vivo* is heterogeneous, and ligaments and tendons are attached to the MTPJ while the Young's modulus of the bone in the first MTPJ remains the same. In this FE model, the ligaments and tendons are ignored.

In addition, further longitudinal studies with larger sample sizes are needed to additionally validate our results and determine the magnitude of the effects of shoe wearing on development and progression of HV.

It can be concluded that stress concentration, stress-shielding, stress shearing and torsional moment generated by positive maximum principal stress and negative minimum principal stress might lead to the first MTPJ bunion. This exemplifies that exercise can promote health and prevent many chronic diseases, but it can also bring injuries. To exercise scientifically with our foot, we need to make sure that our foot bears force in the right way. Shoes change the way our foot bears force. When the way to bear force changes at the MTPJ, the force will lead to HV, suggesting that while footwear can protect our foot, it can also be the reason for some chronic diseases, which cannot be treated by medicine, but by surgery or by wearing simple shoes, or by bare-footed running to rehabilitate. HV may be such a chronic disease. It seems to be the time to respond to Lieberman's call to run bare-footed! This can also shed light on the prevention of other motor organs' injuries.

## Data Availability Statement

The raw data supporting the conclusions of this article will be made available by the authors, without undue reservation.

## Ethics Statement

The studies involving human participants were reviewed and approved by Ethics Committee of Fujian Normal University. The patients/participants provided their written informed consent to participate in this study.

## Author Contributions

YiF, and MD: conceived the study. GY, YuzF, YuxF, RL, YL, and BZ: collected and analyzed data. YiF, MD, DA, PM, GY, YuzF, and ZL: wrote the manuscript. All authors revised the final manuscript.

## Conflict of Interest

The authors declare that the research was conducted in the absence of any commercial or financial relationships that could be construed as a potential conflict of interest.

## Abbreviations

HV: Hallux valgus
MTPJ: Metatarsophalangeal joint
FE: Finite element
PAE: Principal axes of Euler, P1 – P5, Participant 1 – 5
SF: Supplementary File.
