# Supplementary material for: The Role of Footwear in the Pathogenesis of Hallux Valgus: A Proof-of-Concept Finite Element Analysis in Recent Humans and Homo naledi
Source: Front Bioeng Biotechnol. 2020 Jun 30;8:648. doi: 10.3389/fbioe.2020.00648 (PMC7343976; doi:10.3389/fbioe.2020.00648)
Supplement: Supplementary file 1 [file Data_Sheet_1.pdf]

Supplementary Files (SF)

SF Part I Calculating the centroid of bone

Export point-cloud data of bone from 3D reconstruction software (*Mimics*, Materialise, Leuven, Belgium). (x_i, y_i, z_i) refer to the coordinate of point, n the number of point cloud, and (x_c, y_c, z_c) the centroid of the bone and joint (the first metatarsal). The calculating equation of centroid is:

$$\begin{cases} x_c = \frac{1}{n}\sum_{i=0}^{n} x_i \\ y_c = \frac{1}{n}\sum_{i=0}^{n} y_i \\ z_c = \frac{1}{n}\sum_{i=0}^{n} z_i \end{cases} \quad (1).$$

An object has center of mass (COM) and centroid, and a homogeneous object's COM and centroid overlap. (x_m, y_m, z_m) refer to an object's COM, ρ the density. The calculating equation for COM is $x_m = \frac{\sum_{i=0}^{n} x_i\rho}{n\rho} = \frac{\sum_{i=0}^{n} x_i}{n}, y_m = \frac{\sum_{i=0}^{n} y_i\rho}{n\rho} = \frac{\sum_{i=0}^{n} y_i}{n}, z_m = \frac{\sum_{i=0}^{n} z_i\rho}{n\rho} = \frac{\sum_{i=0}^{n} z_i}{n}$, resulting in the overlapping of a homogeneous object's COM and centroid. The COM and centroid of a heterogeneous object differ, but in scanning accuracy, COM and centroid of bone *in vivo* overlap greatly (Fan et al., 2011). In addition, what remains from the bone fossil is the bone's shape. When and ONLY when an object rotates around its COM, an object will not translate. We, therefore, take the bone's centroid as the rotating point of the bone.

Fan, Y., Fan, Y., Li, Z., Loan, M., Lv, C., and Zhang, B. (2011). Optimal principle of bone structure. *PloS One* 6(12).

SF Part II Positioning method of the bone and joint

Rotate the bone (first metatarsal, proximal distal phalanx) around $x \to y \to z$ axes orderly, with its origin at the centroid of the bone.

Rotate the joint (first metatarsal phalange, MTPJ) around $x \to y \to z$ axes orderly, with its origin at the centroid of the first metatarsal.

1) Rotate around axis *x*

We use $oxyz$ to show the spatial rectangular coordinate whose origin is located at centroid of the bone and joint (the first metatarsal). The bone and joint surface consist of finite points. And E_x, E_y, E_z stand for moments of Euler (MoE) (Li et al., 2019) relative to axes x, y, z respectively.

The MoE of the bone and joint are:

$$\begin{cases} E_x = \int (y^2 + z^2) dp \\ E_y = \int (x^2 + z^2) dp \\ E_z = \int (x^2 + y^2) dp \end{cases} \quad (2).$$

The products of Euler of the bone and joint are:

$$\begin{cases} E_{xy} = \int xydp \\ E_{yz} = \int yzdp \\ E_{xz} = \int xzdp \end{cases} \quad (3),$$

where dp stands for the point cloud of bone and joint surface and (x, y, z) for the coordinates of point cloud.

Let the body coordinate system of bone and joint rotate around the axis *x* by α. A new coordinate system $ox_\alpha y_\alpha z_\alpha$ will be generated. The relation between point cloud coordinates $(x_\alpha, y_\alpha, z_\alpha)$ and those of (x, y, z) is:

$$\begin{cases} x_\alpha = x & (a) \\ y_\alpha = y\cos\alpha - z\sin\alpha & (b) \\ z_\alpha = y\sin\alpha + z\cos\alpha & (c) \end{cases} \quad (4).$$

Substitute Eq (4b) and (4c) into $E_x^\alpha = \int (y_\alpha^2 + z_\alpha^2) dp$, MoE relative to axis x, and we will get:

$$\begin{aligned} E_x^\alpha &= \int \left((y\cos\alpha - z\sin\alpha)^2 + (y\sin\alpha + z\cos\alpha)^2 \right) dp \\ &= \int \begin{pmatrix} y^2\cos^2\alpha - 2yz\cos\alpha\sin\alpha + z^2\sin^2\alpha + y^2\sin^2\alpha \\ + 2yz\sin\alpha\cos\alpha + z^2\cos^2\alpha \end{pmatrix} dp \\ &= \int \left(y^2(\cos^2\alpha + \sin^2\alpha) + z^2(\sin^2\alpha + \cos^2\alpha) \right) dp \\ &= \int (y^2 + z^2) dp \\ &= E_x \end{aligned} \quad (5).$$

Eq (5) shows that when rotating around axis *x*, its MoE relative to axis *x* is invariable.

Substitute Eq (4a), (4b) and (4c) into the sum of MoE relative to axis *y* and *z*, i.e. $E_y^\alpha + E_z^\alpha = \int (x_\alpha^2 + z_\alpha^2) dp + \int (x_\alpha^2 + y_\alpha^2) dp$, and we will get:

$$E_y^\alpha + E_z^\alpha = \int x^2 dp + \int (z_\alpha^2 + y_\alpha^2) dp + \int x^2 dp \quad (6).$$

By Eq (5), Eq (6) can be expressed as:

$$\begin{aligned} E_y^\alpha + E_z^\alpha &= \int x^2 dp + \int (z^2 + y^2) dp + \int x^2 dp \\ &= \int (x^2 + z^2) dp + \int (x^2 + y^2) dp \\ &= E_y + E_z \end{aligned} \quad (7).$$

Eq (7) shows that when rotating axis *x*, the sum of MoE relative to axis *y* and *z* is invariable. Together with Eq (5), when rotating axis *x*, the MoE of bone and joint are also invariable.

Substitute Eq (4a) and (4c) into $E_y^\alpha = \int (x_\alpha^2 + z_\alpha^2) dp$, and we will get:

$$\begin{aligned} E_y^\alpha &= \int \left(x^2 + (y\sin\alpha + z\cos\alpha)^2 \right) dp \\ &= \int \left(x^2 + y^2\sin^2\alpha + 2yz\sin\alpha\cos\alpha + z^2\cos^2\alpha \right) dp \end{aligned} \quad (8).$$

Substitute Eq (4a) and (4b) into $I_z^\alpha = \int (x_\alpha^2 + y_\alpha^2) dp$, and we will get:

$$\begin{aligned} E_z^\alpha &= \int \left(x^2 + (y\cos\alpha - z\sin\alpha)^2 \right) dp \\ &= \int \left(x^2 + y^2\cos^2\alpha - 2yz\sin\alpha\cos\alpha + z^2\sin^2\alpha \right) dp \end{aligned} \quad (9).$$

Set up the following equation:

$$f(\alpha, \beta, \gamma)_\alpha = E_y^\alpha - E_z^\alpha \quad (10).$$

By Eq (8) and (9), Eq (10) can be expressed as:

$$f(\alpha, \beta, \gamma)_\alpha = \int \left(y^2(\sin^2\alpha - \cos^2\alpha) + 4yz\sin\alpha\cos\alpha + z^2(\cos^2\alpha - \sin^2\alpha) \right) dp \quad (11).$$

Since $2\sin\alpha\cos\alpha = \sin 2\alpha$, $\cos^2\alpha - \sin^2\alpha = \cos 2\alpha$, Eq (11) can be expressed as:

$$f(\alpha, \beta, \gamma)_\alpha = \int \left(-y^2\cos 2\alpha + 2yz\sin 2\alpha + z^2\cos 2\alpha \right) dp \quad (12).$$

Let

$$\frac{\partial f(\alpha,\beta,\gamma)_\alpha}{\partial\alpha}=0$$

Change Eq (12) to

$$\frac{\partial f(\alpha,\beta,\gamma)_\alpha}{\partial\alpha}=\frac{\partial\left(\int\left(-y^2\cos 2\alpha+2yz\sin 2\alpha+z^2\cos 2\alpha\right)dp\right)}{\partial\alpha} \qquad (13).$$
$$=\int\left(2y^2\sin 2\alpha+4yz\cos 2\alpha-2z^2\sin 2\alpha\right)dp=0$$

Hence,

$$\sin 2\alpha\int y^2dp+2\cos 2\alpha\int yzdp-\sin 2\alpha\int z^2dp=0 \qquad (14).$$

Since

$$\sin 2\alpha\int x^2dp-\sin 2\alpha\int x^2dp=0 \qquad (15).$$

Substitute Eq (15) into Eq (14), and we will get:

$$\sin 2\alpha\int y^2dp+\sin 2\alpha\int x^2dp+2\cos 2\alpha\int yzdp-\sin 2\alpha\int z^2dp-\sin 2\alpha\int x^2dp=0 \qquad (16).$$

By Eq (2), Eq (16) can be expressed as:

$$\sin 2\alpha E_z+2\cos 2\alpha E_{yz}-\sin 2\alpha E_y=0 \qquad (17).$$

Divide both sides of Eq 17 by $\cos 2\alpha$, and we will get:

$$\tan 2\alpha E_z+2E_{yz}-\tan 2\alpha E_y=0 \qquad (18).$$

Next, we will get:

$$\tan 2\alpha=\frac{2E_{yz}}{E_y-E_z} \qquad (19).$$

Then, get the inverse function of Eq (19):

$$\alpha=\frac{1}{2}\arctan\left(\frac{2E_{yz}}{E_y-E_z}\right) \qquad (20).$$

2) Rotate around axis *y*

After rotating around axis *x* by α, $ox_\alpha y_\alpha z_\alpha$ are used to stand for the spatial rectangular coordinate system of bone and joint with the origin locating at the centroid of the bone and joint (the first metatarsal). $E_{xx}^\alpha, E_{yy}^\alpha, E_{zz}^\alpha$ stand for the MoE of axes $x_\alpha, y_\alpha, z_\alpha$ respectively.

The MoE of the bone and joint are:

$$\begin{cases}E_{xx}^\alpha=\int\left(y_\alpha^2+z_\alpha^2\right)dp\\ E_{yy}^\alpha=\int\left(x_\alpha^2+z_\alpha^2\right)dp\\ E_{zz}^\alpha=\int\left(x_\alpha^2+y_\alpha^2\right)dp\end{cases} \qquad (21).$$

The products of Euler of the bone and joint are:

$$\begin{cases}E_{xy}^\alpha=\int x_\alpha y_\alpha dp\\ E_{yz}^\alpha=\int y_\alpha z_\alpha dp\\ E_{xz}^\alpha=\int x_\alpha z_\alpha dp\end{cases} \qquad (22),$$

where *dp* stands for the point cloud of bone and joint surface and $(x_\alpha, y_\alpha, z_\alpha)$ for the coordinate of point cloud.

Let the body coordinate system of the bone and joint rotate around the axis y by β. A new coordinate system $ox_{\alpha\beta}y_{\alpha\beta}z_{\alpha\beta}$ will be generated. The relation between point cloud coordinates $(x_{\alpha\beta}, y_{\alpha\beta}, z_{\alpha\beta})$ and those of $(x_\alpha, y_\alpha, z_\alpha)$ is:

$$\begin{cases} x_{\alpha\beta} = x_\alpha \cos\beta + z_\alpha \sin\beta & (a) \\ y_{\alpha\beta} = y_\alpha & (b) \\ z_{\alpha\beta} = -x_\alpha \sin\beta + z_\alpha \cos\beta & (c) \end{cases} \qquad (23).$$

Substitute Eq (23a) and (23c) into the Eq $E_{yy}^{\alpha\beta} = \int \left(x_{\alpha\beta}^2 + z_{\alpha\beta}^2\right) dp$, the MoE relative to axis x, and we will get:

$$\begin{aligned} E_{yy}^{\alpha\beta} &= \int \left((x_\alpha \cos\beta + z_\alpha \sin\beta)^2 + (-x_\alpha \sin\beta + z_\alpha \cos\beta)^2 \right) dp \\ &= \int \begin{pmatrix} x_\alpha^2 \cos^2\beta + 2x_\alpha z_\alpha \cos\beta \sin\beta + z_\alpha^2 \sin^2\beta + x_\alpha^2 \sin^2\beta \\ -2x_\alpha z_\alpha \sin\beta \cos\beta + z_\alpha^2 \cos^2\beta \end{pmatrix} dp \\ &= \int \left(x_\alpha^2 (\cos^2\beta + \sin^2\beta) + z_\alpha^2 (\sin^2\beta + \cos^2\beta) \right) dp \qquad (24). \\ &= \int \left(x_\alpha^2 + z_\alpha^2 \right) dp \\ &= E_{yy}^{\alpha} \end{aligned}$$

Eq (24) shows that when rotating around axis y, its MoE relative to axis y is invariable.

Substitute Eq (23a), (23b) and (23c) into the sum of MoE relative to axis x and z, i.e. $E_{xx}^{\alpha\beta} + E_{zz}^{\alpha\beta} = \int \left(y_{\alpha\beta}^2 + z_{\alpha\beta}^2\right) dp + \int \left(x_{\alpha\beta}^2 + y_{\alpha\beta}^2\right) dp$, and we will get:

$$E_{xx}^{\alpha\beta} + E_{zz}^{\alpha\beta} = \int y_\alpha^2 dp + \int \left(x_{\alpha\beta}^2 + z_{\alpha\beta}^2\right) dp + \int y_\alpha^2 dp \qquad (25).$$

By Eq (24), Eq (25) can be expressed as:

$$\begin{aligned} E_{xx}^{\alpha\beta} + E_{zz}^{\alpha\beta} &= \int y_\alpha^2 dp + \int \left(x_\alpha^2 + z_\alpha^2\right) dp + \int y_\alpha^2 dp \\ &= \int \left(y_\alpha^2 + z_\alpha^2\right) dp + \int \left(x_\alpha^2 + z_\alpha^2\right) dp \qquad (26). \\ &= E_{xx}^{\alpha} + E_{zz}^{\alpha} \end{aligned}$$

Eq (26) shows that by rotating axis y, the MoE relative to axis x and z are invariable. Together with Eq (24), by rotating around axis y, the Euler of the bone and joint is also invariable.

Substitute Eq (23b) and (23c) into $E_{xx}^{\alpha\beta} = \int \left(y_{\alpha\beta}^2 + z_{\alpha\beta}^2\right) dp$, and we will get:

$$\begin{aligned} E_{xx}^{\alpha\beta} &= \int \left(y_{\alpha\beta}^2 + (-x_\alpha \sin\beta + z_\alpha \cos\beta)^2 \right) dp \\ &= \int \left(y_\alpha^2 + x_\alpha^2 \sin^2\beta - 2x_\alpha z_\alpha \sin\beta \cos\beta + z_\alpha^2 \cos^2\beta \right) dp \end{aligned} \qquad (27).$$

Substitute Eq (23a) and (23b) into $E_{zz}^{\alpha\beta} = \int \left(x_{\alpha\beta}^2 + y_{\alpha\beta}^2\right) dp$, and we will get:

$$\begin{aligned} E_{zz}{}^{\alpha\beta} &= \int \left((x_\alpha \cos\beta + z_\alpha \sin\beta)^2 + y_\alpha^2 \right) dp \\ &= \int \left(x_\alpha^2 \cos^2\beta + 2x_\alpha z_\alpha \sin\beta \cos\beta + z_\alpha^2 \sin^2\beta + y_\alpha^2 \right) dp \end{aligned} \qquad (28).$$

Set up the following equation:

$$f(\alpha,\beta,\gamma)_\beta = E_{zz}^{\alpha\beta} - E_{xx}^{\alpha\beta} \qquad (29).$$

By Eq (27) and (28), Eq (29) can be expressed as:

$$f(\alpha,\beta,\gamma)_\beta = \int \left(x_\alpha^2 (\cos^2\beta - \sin^2\beta) + 4x_\alpha z_\alpha \sin\beta \cos\beta - z_\alpha^2 (\cos^2\beta - \sin^2\beta) \right) dp \quad (30).$$

Since $2\sin\beta\cos\beta = \sin 2\beta$, $\cos^2\beta - \sin^2\beta = \cos 2\beta$, Eq (30) can be expressed as:

$$f(\alpha,\beta,\gamma)_\beta = \int \left(x_\alpha^2 \cos 2\beta + 2x_\alpha z_\alpha \sin 2\beta - z_\alpha^2 \cos 2\beta \right) dp \qquad (31).$$

Let

$$\frac{\partial f(\alpha,\beta,\gamma)_\beta}{\partial \beta} = 0.$$

Since

$$\frac{\partial f(\alpha,\beta,\gamma)_\beta}{\partial \beta} = \frac{\partial \left(\int \left(x_\alpha^2 \cos 2\beta + 2x_\alpha z_\alpha \sin 2\beta - z_\alpha^2 \cos 2\beta \right) dp \right)}{\partial \beta}$$

$$= \int \left(-2x_\alpha^2 \sin 2\beta + 4x_\alpha z_\alpha \cos 2\beta + 2z_\alpha^2 \sin 2\beta \right) dp \qquad (32).$$

Hence

$$-\sin 2\beta \int x_\alpha^2 dp + 2\cos 2\beta \int x_\alpha z_\alpha dp + \sin 2\beta \int z_\alpha^2 dp = 0 \qquad (33).$$

Since

$$\sin 2\beta \int y_\alpha^2 dp - \sin 2\beta \int y_\alpha^2 dp = 0 \qquad (34).$$

Substitute Eq (34) into Eq (33), and we will get:

$$-\sin 2\beta \int x_\alpha^2 dp - \sin 2\beta \int y_\alpha^2 dp + 2\cos 2\beta \int x_\alpha z_\alpha dp + \sin 2\alpha \int z_\alpha^2 dp + \sin 2\alpha \int y_\alpha^2 dp = 0 \qquad (35).$$

By Eq (21), Eq (35) can be expressed as:

$$-\sin 2\beta E_{zz}^\alpha + 2\cos 2\beta E_{xz}^\alpha + \sin 2\beta E_{xx}^\alpha = 0 \qquad (36).$$

Divide both sides of Eq (36) by $\cos 2\beta$, and we will get:

$$-\tan 2\beta E_{zz}^\alpha + 2\beta E_{xz}^\alpha + \tan 2\beta E_{xx}^\alpha = 0 \qquad (37).$$

Next, we will get:

$$\tan 2\beta = -\frac{2E_{xz}^\alpha}{E_{xx}^\alpha - E_{zz}^\alpha} \qquad (38).$$

Then, get the inverse function of Eq (38):

$$\beta = -\frac{1}{2}\arctan\left(\frac{2E_{xz}^\alpha}{E_{xx}^\alpha - E_{zz}^\alpha} \right) \qquad (39).$$

3) To rotate around axis *z*

After rotating around axis *x* by α and around axis *y* by β, $ox_{\alpha\beta}y_{\alpha\beta}z_{\alpha\beta}$ are used to stand for the spatial rectangular coordinate system of the bone and joint with the origin locating at the centroid of the bone and joint (the first metatarsal). $E_{xx}^{\alpha\beta}, E_{yy}^{\alpha\beta}, E_{zz}^{\alpha\beta}$ stand for the MoE of axes $x_{\alpha\beta}, y_{\alpha\beta}, z_{\alpha\beta}$ respectively.

The MoE of the bone and joint will be:

$$\begin{cases} E_{xx}^{\alpha\beta} = \int \left(y_{\alpha\beta}^2 + z_{\alpha\beta}^2 \right) dp \\ E_{yy}^{\alpha\beta} = \int \left(x_{\alpha\beta}^2 + z_{\alpha\beta}^2 \right) dp \\ E_{zz}^{\alpha\beta} = \int \left(x_{\alpha\beta}^2 + y_{\alpha\beta}^2 \right) dp \end{cases} \qquad (40).$$

The products of Euler of the bone and joint will be:

$$\begin{cases} E_{xy}^{\alpha\beta} = \int x_{\alpha\beta} y_{\alpha\beta} dp \\ E_{yz}^{\alpha\beta} = \int y_{\alpha\beta} z_{\alpha\beta} dp \\ E_{xz}^{\alpha\beta} = \int x_{\alpha\beta} z_{\alpha\beta} dp \end{cases} \qquad (41),$$

where dp stands for the point cloud of bone and joint surface and $\left(x_{\alpha\beta}, y_{\alpha\beta}, z_{\alpha\beta} \right)$ for the coordinate of point cloud.

Let the body coordinate system of the bone and joint rotate around the axis *z* by γ. A new coordinate system $ox_{\alpha\beta\gamma}y_{\alpha\beta\gamma}z_{\alpha\beta\gamma}$ will be formed. The relation between point cloud coordinates $\left(x_{\alpha\beta\gamma}, y_{\alpha\beta\gamma}, z_{\alpha\beta\gamma} \right)$ and those of $\left(x_{\alpha\beta}, y_{\alpha\beta}, z_{\alpha\beta} \right)$ is:

$$\begin{cases} x_{\alpha\beta\gamma} = x_{\alpha\beta}\cos\gamma - y_{\alpha\beta}\sin\gamma & (a) \\ y_{\alpha\beta\gamma} = x_{\alpha\beta}\sin\gamma + y_{\alpha\beta}\cos\gamma & (b) \\ z_{\alpha\beta\gamma} = z_{\alpha\beta} & (c) \end{cases} \qquad (42).$$

Substitute Eq (42a) and (42b) into the Eq $E_{zz}^{\alpha\beta\gamma} = \int \left(x_{\alpha\beta\gamma}^2 + y_{\alpha\beta\gamma}^2\right)dp$, the MoE relative to axis *z*, and we will get:

$$\begin{aligned} E_{zz}^{\alpha\beta\gamma} &= \int \left(\left(x_{\alpha\beta}\cos\gamma - y_{\alpha\beta}\sin\gamma\right)^2 + \left(x_{\alpha\beta}\sin\gamma + y_{\alpha\beta}\cos\gamma\right)^2 \right)dp \\ &= \int \begin{pmatrix} x_{\alpha\beta}^2\cos^2\gamma - 2x_{\alpha\beta}y_{\alpha\beta}\cos\gamma\sin\gamma + y_{\alpha\beta}^2\sin^2\gamma + x_{\alpha\beta}^2\sin^2\gamma \\ + 2x_{\alpha\beta}y_{\alpha\beta}\sin\gamma\cos\gamma + y_{\alpha\beta}^2\cos^2\gamma \end{pmatrix} dp \\ &= \int \left(x_{\alpha\beta}^2\left(\cos^2\gamma + \sin^2\gamma\right) + y_{\alpha\beta}^2\left(\sin^2\gamma + \cos^2\gamma\right)\right)dp \qquad (43). \\ &= \int \left(x_{\alpha\beta}^2 + y_{\alpha\beta}^2\right)dp \\ &= E_{zz}^{\alpha\beta} \end{aligned}$$

Eq (43) shows that when rotating around axis *z*, its MoE relative to axis *z* is invariable.

Substitute Eq (42a), (42b) and (42c) into the sum of MoE relative to axis *x* and *y*, i.e. $E_{xx}^{\alpha\beta\gamma} + E_{yy}^{\alpha\beta\gamma} = \int \left(y_{\alpha\beta\gamma}^2 + z_{\alpha\beta\gamma}^2\right)dp + \int \left(x_{\alpha\beta\gamma}^2 + z_{\alpha\beta\gamma}^2\right)dp$, and we will get:

$$\begin{aligned} E_{xx}^{\alpha\beta\gamma} + E_{yy}^{\alpha\beta\gamma} &= \int z_{\alpha\beta}^2 dp + \int \left(x_{\alpha\beta\gamma}^2 + y_{\alpha\beta\gamma}^2\right)dp + \int z_{\alpha\beta}^2 dp \\ &= \int z_{\alpha\beta}^2 dp + \int \left(x_{\alpha\beta}^2 + y_{\alpha\beta}^2\right)dp + \int z_{\alpha\beta}^2 dp \qquad (44). \\ &= \int \left(y_{\alpha\beta}^2 + z_{\alpha\beta}^2\right)dp + \int \left(x_{\alpha\beta}^2 + z_{\alpha\beta}^2\right)dp \\ &= E_{xx}^{\alpha\beta} + E_{yy}^{\alpha\beta} \end{aligned}$$

Eq (44) shows that by rotating axis *z*, the sum of MoE relative to axis *x* and *y* is invariable. Together with Eq (43), by rotating axis *z*, the Euler of the bone and joint is invariable.

Substitute Eq (42b) and (42c) into Eq $E_{xx}^{\alpha\beta\gamma} = \int \left(y_{\alpha\beta\gamma}^2 + z_{\alpha\beta\gamma}^2\right)dp$, and we will get:

$$\begin{aligned} E_{xx}^{\alpha\beta\gamma} &= \int \left(\left(x_{\alpha\beta}\sin\gamma + y_{\alpha\beta}\cos\gamma\right)^2 + z_{\alpha\beta}^2 \right)dp \\ &= \int \left(x_{\alpha\beta}^2\sin^2\gamma + 2x_{\alpha\beta}y_{\alpha\beta}\sin\gamma\cos\gamma + y_{\alpha\beta}^2\cos^2\gamma + z_{\alpha\beta}^2 \right)dp \end{aligned} \qquad (45).$$

Substitute Eq (42a) and (42c) into $E_{yy}^{\alpha\beta\gamma} = \int \left(x_{\alpha\beta\gamma}^2 + z_{\alpha\beta\gamma}^2\right)dp$, and we will get:

$$\begin{aligned} E_{yy}^{\alpha\beta\gamma} &= \int \left(\left(x_{\alpha\beta}\cos\gamma - y_{\alpha\beta}\sin\gamma\right)^2 + z_{\alpha\beta}^2 \right)dp \\ &= \int \left(x_{\alpha\beta}^2\cos^2\gamma - 2x_{\alpha\beta}y_{\alpha\beta}\sin\gamma\cos\gamma + y_{\alpha\beta}^2\sin^2\gamma + z_{\alpha\beta}^2 \right)dp \end{aligned} \qquad (46).$$

Set up the following equation:

$$f(\alpha,\beta,\gamma)_\gamma = E_{xx}^{\alpha\beta\gamma} - E_{yy}^{\alpha\beta\gamma} \qquad (47).$$

By Eq (45) and (46), Eq (47) can be expressed as:

$$f(\alpha,\beta,\gamma)_\gamma = \int \left(-x_{\alpha\beta}^2\left(\cos^2\gamma - \sin^2\gamma\right) + 4x_{\alpha\beta}y_{\alpha\beta}\sin\gamma\cos\gamma + y_{\alpha\beta}^2\left(\cos^2\gamma - \sin^2\gamma\right)\right)dp \qquad (48)$$

Since $2\sin\gamma\cos\lambda = \sin 2\gamma$, $\cos^2\gamma - \sin^2\gamma = \cos 2\gamma$, Eq (48) can be expressed as:

$$f(\alpha,\beta,\gamma)_\gamma = \int \left(-x_{\alpha\beta}^2\cos 2\gamma + 2x_{\alpha\beta}y_{\alpha\beta}\sin 2\gamma + y_{\alpha\beta}^2\cos 2\gamma \right)dp \qquad (49).$$

Let

$$\frac{\partial f(\alpha,\beta,\gamma)_\gamma}{\partial\gamma} = 0$$

Since

$$\frac{\partial f(\alpha,\beta,\gamma)_\gamma}{\partial\gamma}=\frac{\partial\left(\int\left(-x_{\alpha\beta}^2\cos 2\gamma+2x_{\alpha\beta}y_{\alpha\beta}\sin 2\gamma+y_{\alpha\beta}^2\cos 2\gamma\right)dp\right)}{\partial\gamma} \quad (50).$$

$$=\int\left(2x_{\alpha\beta}^2\sin 2\gamma+4x_{\alpha\beta}y_{\alpha\beta}\cos 2\gamma-2y_{\alpha\beta}^2\cos 2\gamma\right)dp$$

Hence

$$\sin 2\gamma\int x_{\alpha\beta}^2 dp+2\cos 2\gamma\int x_{\alpha\beta}y_{\alpha\beta}dp-\sin 2\gamma\int y_{\alpha\beta}^2 dp=0 \quad (51).$$

Since

$$\sin 2\gamma\int z_{\alpha\beta}^2 dp-\sin 2\gamma\int z_{\alpha\beta}^2 dp=0 \quad (52).$$

Substitute Eq (52) into Eq (51), and we will get:

$$\sin 2\gamma\int x_{\alpha\beta}^2 dp+\sin 2\gamma\int z_{\alpha\beta}^2 dp+2\cos 2\gamma\int x_{\alpha\beta}y_{\alpha\beta}dp-\sin 2\gamma\int y_{\alpha\beta}^2 dp-\sin 2\gamma\int z_{\alpha\beta}^2 dp=0 \quad (53).$$

By Eq (40) and (9), Eq (53) can be expressed as:

$$\sin 2\gamma E_{yy}^{\alpha\beta}+2\cos 2\gamma E_{xy}^{\alpha\beta}-\sin 2\gamma E_{xx}^{\alpha\beta}=0 \quad (54).$$

Divide both sides of Eq (54) by $\cos 2\gamma$, and we will get:

$$\tan 2\gamma E_{yy}^{\alpha\beta}+2E_{xy}^{\alpha\beta}-\tan 2\gamma E_{xx}^{\alpha\beta}=0 \quad (55).$$

Next, we will get:

$$\tan 2\gamma=\frac{2E_{xy}^{\alpha\beta}}{E_{xx}^{\alpha\beta}-E_{yy}^{\alpha\beta}} \quad (56).$$

Then, get the inverse function of Eq (56):

$$\gamma=\frac{1}{2}\arctan\left(\frac{2E_{xy}^{\alpha\beta}}{E_{xx}^{\alpha\beta}-E_{yy}^{\alpha\beta}}\right) \quad (57).$$

When all the products of Euler become nil, the bone and joint rotation is complete. And then rotate the bone and joint using the same method until all its products of Euler become nil. After that, rotate the bone and joint in reverse order of how athlete bones rotate.

Li, R., Fan, Y., Liu, Y., Antonijevic, Đ., Li, Z., and Djuric, M. (2019). Homo naledi did not have flat foot. *Homo Int. Z. Vgl. Forsch. Am. Menschen.* 70, 139–146.

SF Part III Positioning procedure of the first metatarsal, proximal phalanx and distal phalanx

Use Eq (1) to calculate the centroid of the first metatarsal, proximal phalanx and distal phalanx, respectively. Translate the bone's centroid to $(-x_o, -y_o, -z_o)$ so that the corresponding centroid will be $(0.00, 0.00, 0.00)$, with the bone's centroid as the rotating point. Use Eq (20) to calculate the angle of bone rotating around axis x. Calculate the corresponding result by Eq (36) and obtain the angle of rotating around axis y. Calculate the corresponding result by Eq (1) and obtain the angle of rotating around axis z. Then calculate the corresponding result by Eqs (21), (37) and (1) and obtain the angles of rotating around axes x_1, y_1 and z_1 accordingly and when the corresponding result calculated by Eq (21) and the value of rotating around axis x_2 is zero, the positioning is done. See Table S1 for the positioning result from P1's first-time scanning of the first metatarsal, proximal phalanx and distal phalanx.

Table S1 Positioning angles of the first metatarsal, proximal phalanx and distal phalanx around axes *x, y, z,* *x₁, y₁, and z₁.*

Item	axis x	axis y	axis z	axis x_1	axis y_1	axis z_1
First metatarsal	34.53	-9.84	29.30	0.11	0.06	0.00
Proximal phalanx	44.63	-36.04	31.35	-2.54	-1.84	-0.42
Distal phalanx	40.77	38.20	9.96	34.68	-1.35	-0.69

See Figure S1 for the rotation procedure.

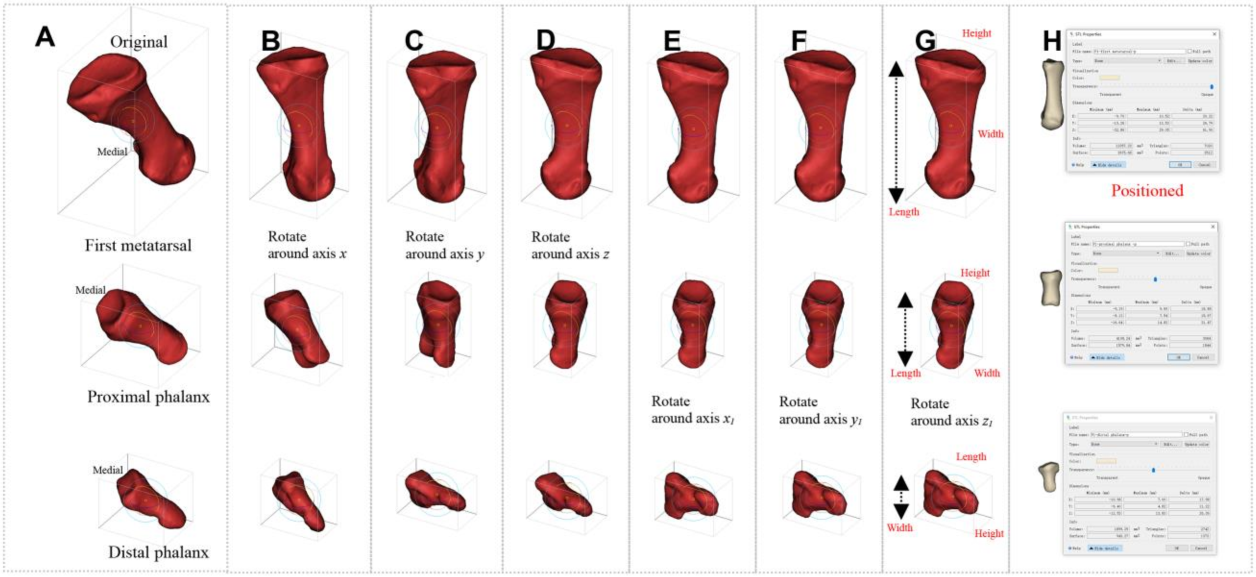

Figure S1 Positioning procedure of the first metatarsal, proximal phalanx and distal phalanx **(A)** Original (scanning) posture. **(B)-(G)** Rotating around x, y, z, x_1, y_1 and z_1, respectively. When rotating around x_2, with an angle of zero, the positioning is accomplished.

After the first metatarsal, proximal phalanx and distal phalanx were positioned, the bones' properties were shown by the software (*Mimics*). See Figure S1 **(H).** The detailed information of the point's coordinate from bones' three principal axes and the length, width and height based on bone's body coordinate system were shown in Table S2.

Table S2 Bone's properties (Unit: mm)

	First metatarsal				Proximal phalanx				Distal phalanx			
	x	y	z	Delta	x	y	z	Delta	x	y	z	Delta
Point 1 (axis x)	10.52	0.00	0.00	20.22	9.69	0.00	0.00	18.88	7.00	0.00	0.00	17.98
Point 2 (axis x)	-9.70	0.00	0.00		-9.19	0.00	0.00		-10.98	0.00	0.00	
Point 1 (axis y)	0.00	13.53	0.00	26.79	0.00	7.54	0.00	15.67	0.00	4.82	0.00	11.22
Point 2 (axis y)	0.00	-13.26	0.00		0.00	-8.13	0.00		0.00	-6.40	0.00	
Point 1 (axis z)	0.00	0.00	29.05	61.90	0.00	0.00	14.83	31.47	0.00	0.00	13.83	26.36
Point 2 (axis z)	0.00	0.00	-32.86		0.00	0.00	-16.64		0.00	0.00	-12.53	

SF Part IV Positioning procedure of the body coordinate of the first MTPJ

Use Eq (1) to calculate the centroid of the first MTPJ. Translate the first MTPJ's centroid to the bone's centroid till $(-x_o, -y_o, -z_o)$, with the first metatarsal's centroid as the rotating point. Use Eq (20) to calculate P1'sfirst metatarsal from the first-time scan and obtain the angle of rotating around axis x to be 34.53°. Calculate the corresponding result by Eq (36) and obtain the angle of rotating around axis y to be-9.84°. Calculate the corresponding result by Eq (57) and obtain the angle of rotating around axis z to be29.30°. Then calculate the corresponding result by Eqs (20), (36) and (57) and obtain the angles of rotating around axes x_1,$y_1$and $z_1$to be 0.11°, 0.06°, and 0.00°accordingly and when the corresponding result calculated by Eq (20) and the value of rotating around axis x_2 is zero, the positioning is done. See Figure S2 for the specific results.

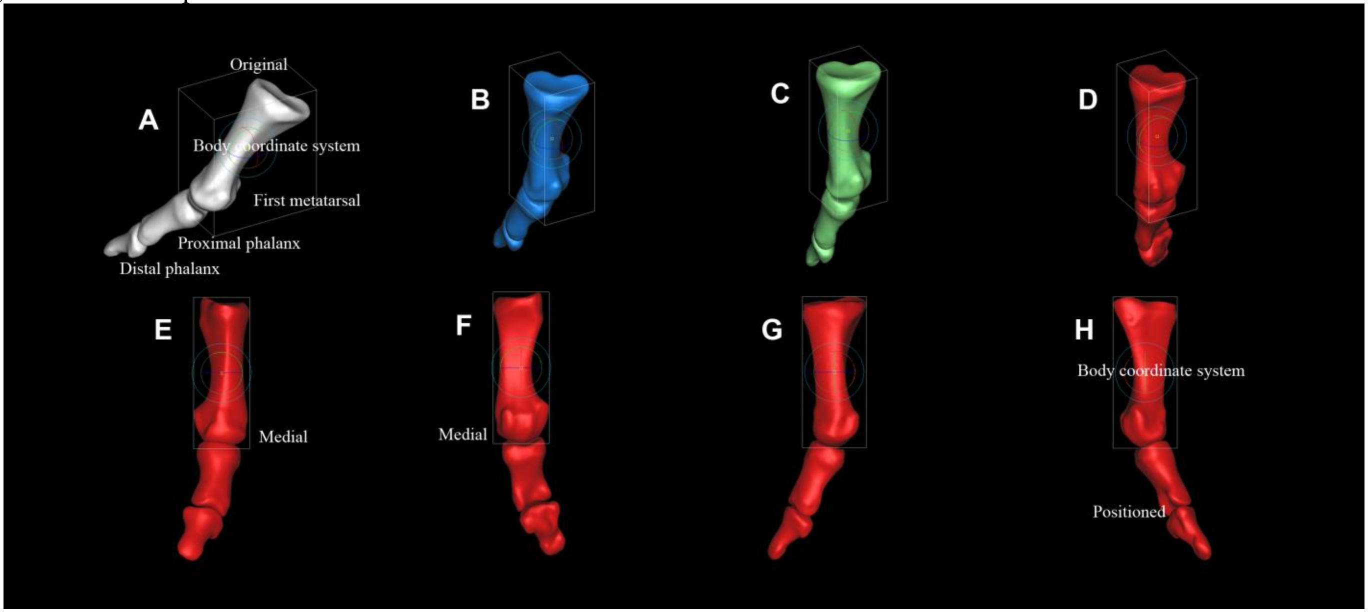

Figure S2 Positioning procedure of the first MTPJ's body coordinate. **(A)** P1's first MTPJ body coordinate from the first-time scan. **(B)-(F)** Rotate around axis x, y, z, x_1, and y_1. **(G)** Rotate around axis z_1 till the value reaches 0.00°. **(H)** P1's first MTPJ body coordinate is accomplished.

Original (scanning) posture and positioned posture of the first MTPJ

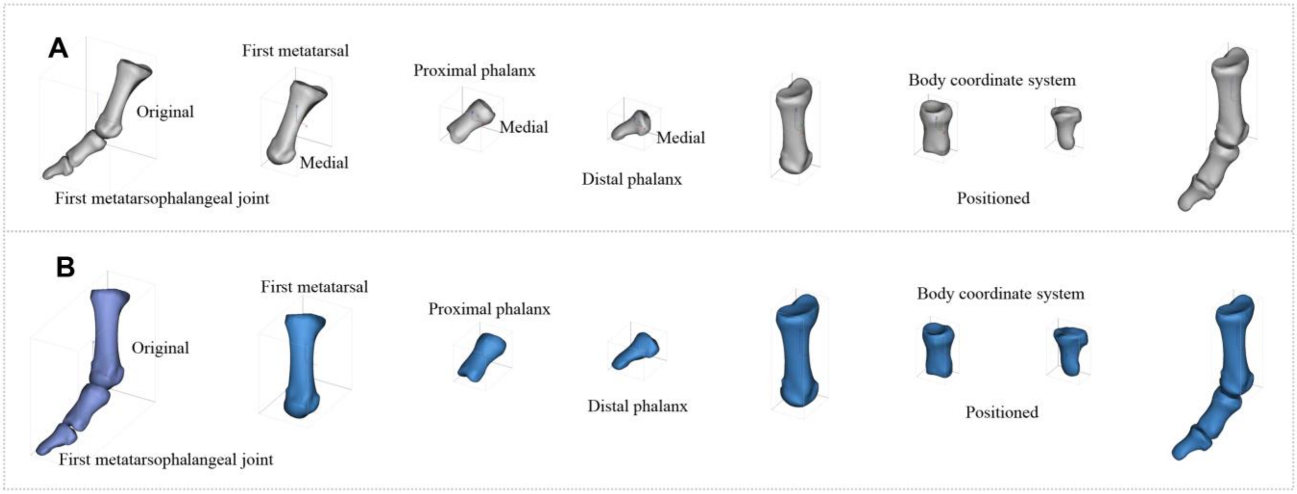

Figure S3 Scanning posture (original) and positioned posture of the first MTPJ from **(A)** the first-time scanning and **(B)** the second-time scanning.

SF Part V Standardizing the geometric model

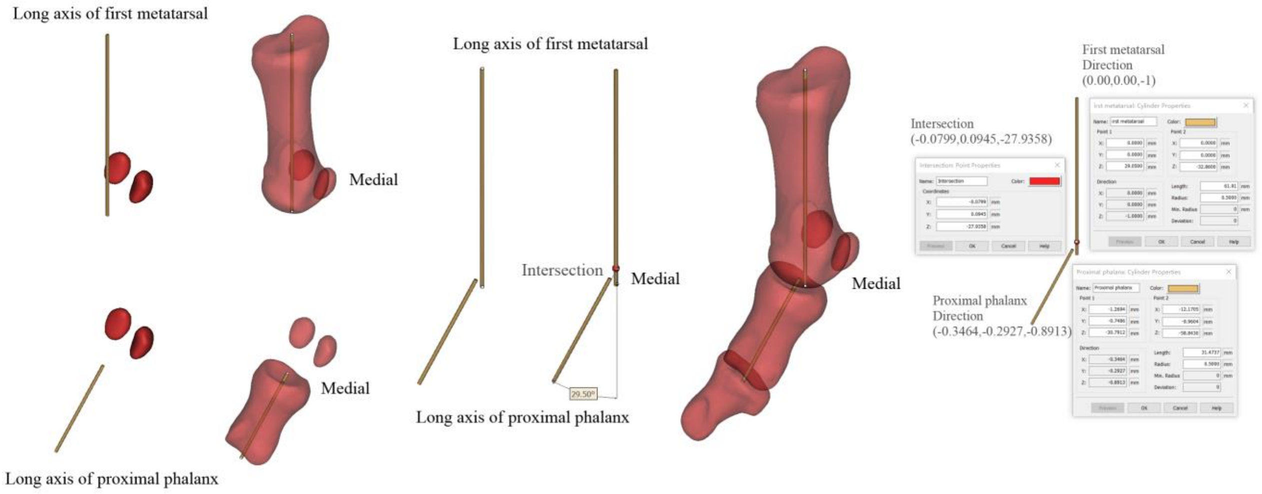

Figure S4 Standardizing the first MTPJ geometric model. **(A)-(E)** P1 – P5, respectively.

SF Part VI Calculating the first MTPJ angle

$p_1 = (x_1, y_1, z_1)$refers to the first metatarsal's long axis proximal coordinate point. Via Table S2, P1's proximal coordinate point is$p_1 = (0.00,0.00,29.05)$.$p_2 = (x_2, y_2, z_2)$refers to the first metatarsal's long axis distal coordinate point. Via Table S2, P1's distal coordinate point is $p_2 = (0.00,0.00,-32.86)$. Use $p_1, p_2$to draw P1's first metatarsal long axis.

$p_3 = (x_3, y_3, z_3)$refers to the proximal phalanx's long axis proximal coordinate point. Via Table S2, P1's proximal coordinate point is$p_3 = (0.00,0.00,14.83)$.$p_4 = (x_4, y_4, z_4)$refers to the proximal phalanx's long axis distal coordinate point, Via Table S2, P1's distal coordinate point is$p_4 = (0.00,0.00,-16.64)$. Use $p_3, p_4$to draw P1's proximal phalanx long axis.

Use Eq (1) to calculate the centroid of the proximal phalanx in Figure S2, taking the centroid of the proximal phalanx as the rotating point. According to Literature (Fan et al., 2019) shows that p_3, p_4 rotate around axis *z* with an angle of 0.42°, around axis *y* of 1.84°, and axis *x* of 2.54°, respectively. Then rotate around axis *z* of -31.35°, axis *y* of 36.04° and axis *x* of -44.63°. Now, P1's distal phalanx long axis proximal coordinate point is $p_5 = (-1.27, -0.75, -30.79)$, and the distal one is $p_6 = (-12.17, -9.96, -58.84)$.

We observed from *Mimics* software system that the direction of the positioned first metatarsal long axis is $(0.00,0.00,-1)$, the length 61.91mm while the direction of the proximal phalanx long axis is $(0.00,0.00,-1)$ and the length 31.47mm. After the rotation, the direction is $(-0.35,-0.29,-0.89)$, and the length 31.47mm.

Set the direction of the first metatarsal long axis as (x_1, y_1, z_1), and that of the proximal phalanx long axis as (x_2, y_2, z_2). Then, the angle between the two will be:

$$\theta = \arccos\left(\frac{x_1x_2 + y_1y_2 + z_1z_2}{\sqrt{x_1^2 + y_1^2 + z_1^2} \times \sqrt{x_2^2 + y_2^2 + z_2^2}}\right) \qquad (58)$$

Use Eq (58) to position the angle between the direction of the first metatarsal long axis $(0.00,0.00,-1)$ and that of the rotated proximal phalanx long axis $(-0.35,-0.29,-0.89)$ to be 26.97°. See Figure S4 for details.

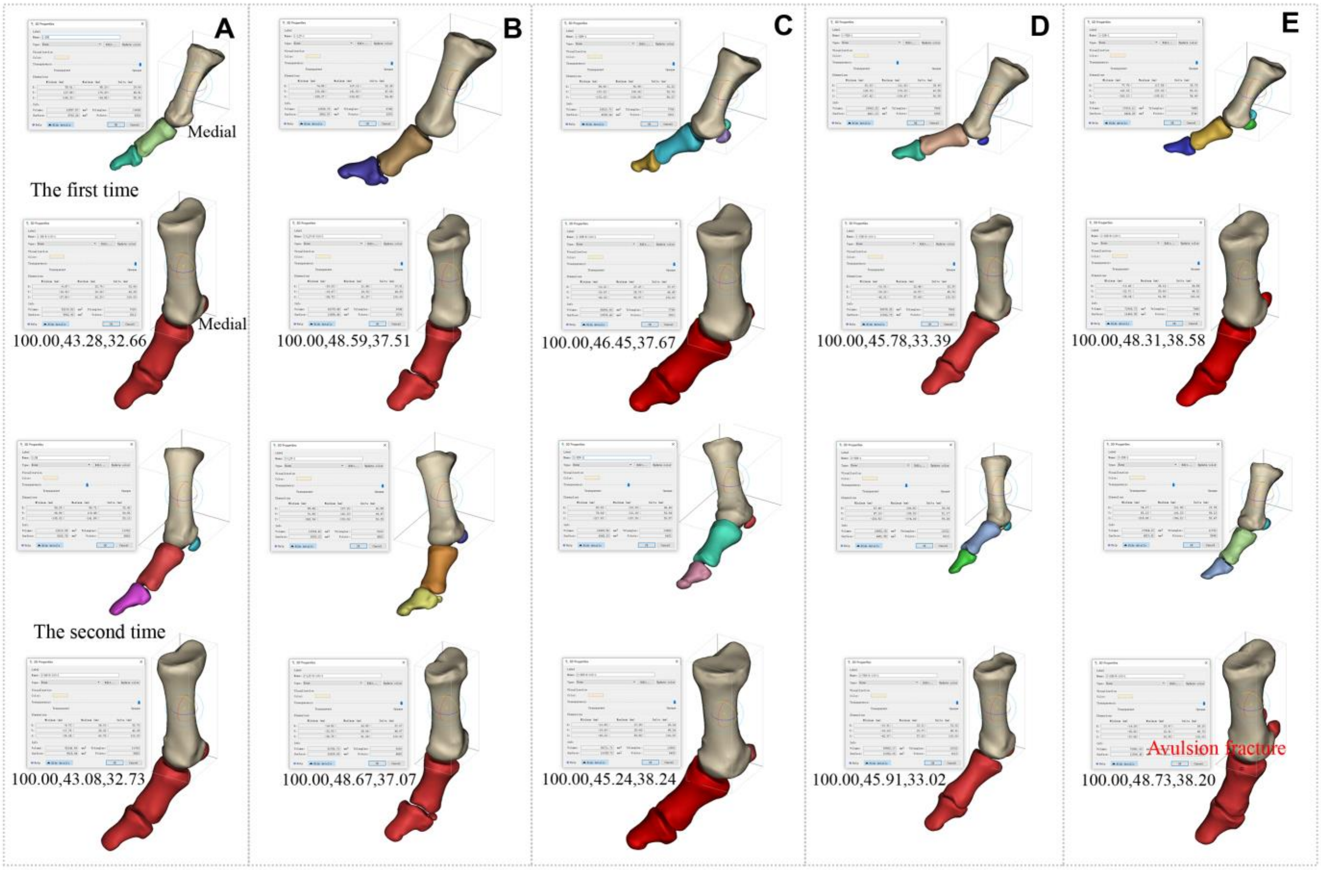

Figure S5 Measurement of P1's first MTPJ angle (between long axes of the first metatarsal and the proximal phalanx).

Fan, Y., Antonijevic, D., Antic, S., Li, R., Liu, Y., Li, Z., et al. (2019). Reconstructing the First Metatarsophalangeal Joint of Homo naledi. *Front. Bioeng. Biotechnol*. 7, 167.

SF Part VII Positioning the phalanx posture

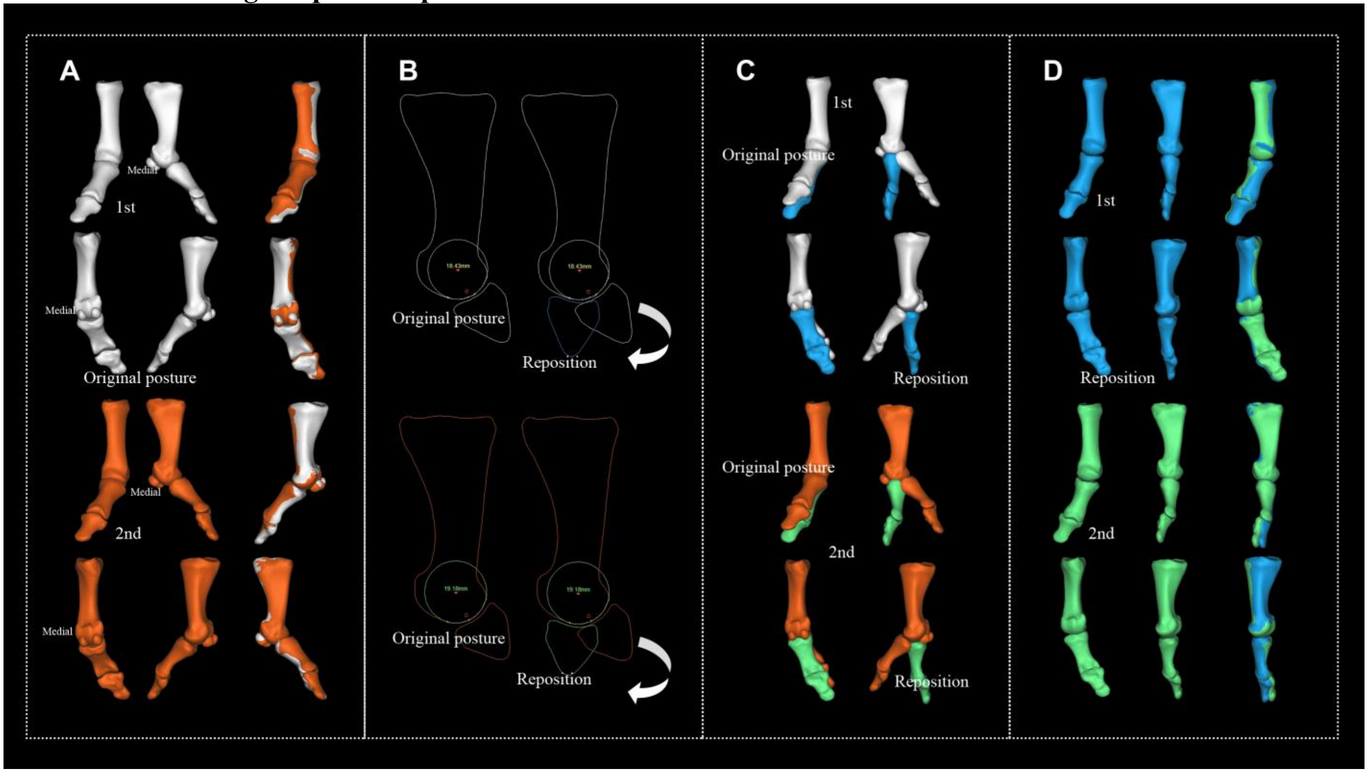

Figure S6 Positioning the phalanx posture of P4. **(A)** Reconstruction of P4's first MTPJ. **(B)** Standardization of P4's phalanx posture relative to metatarsal in coronal plane. **(C)** The first MTPJ of P4 before being positioned. **(D)** The first MTPJ of P4 after being positioned.

SF Part VIII Structure transformation of the first MTPJ

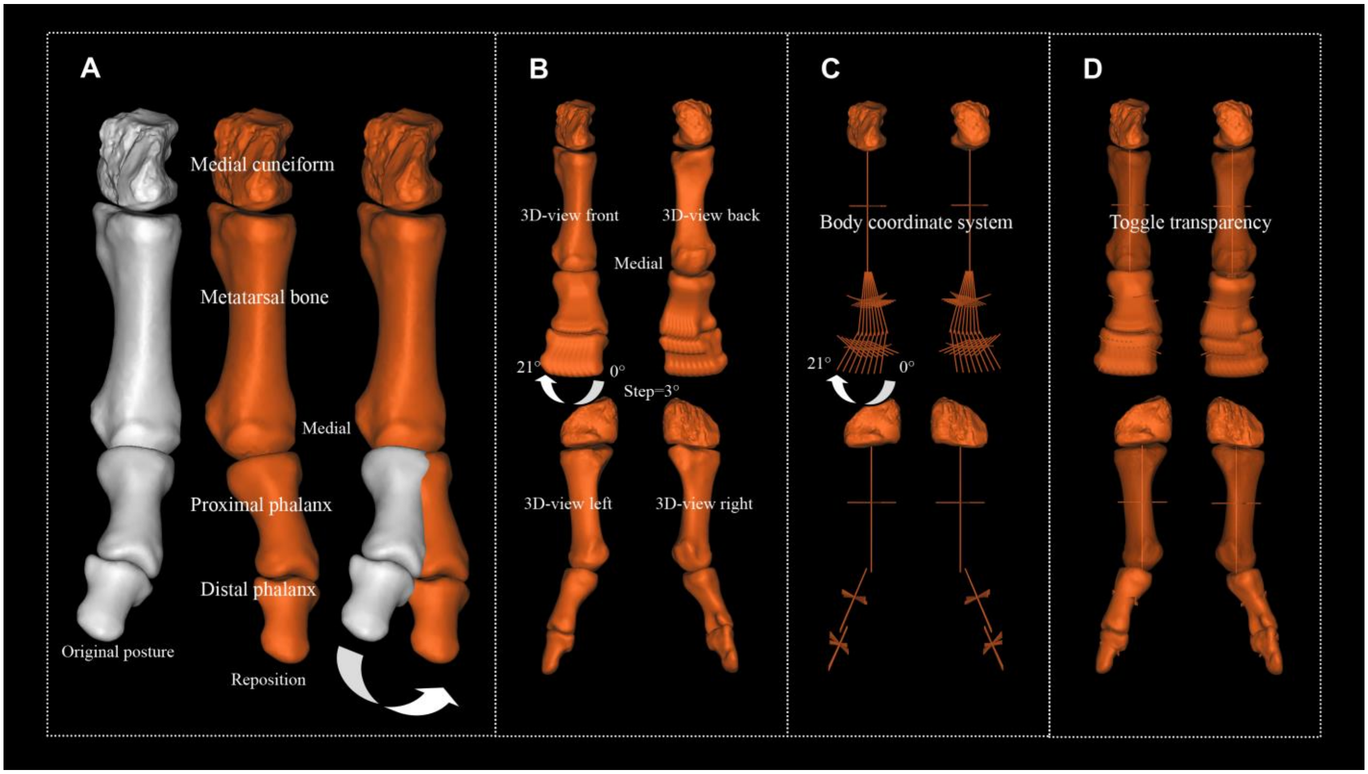

Figure S7 Structure transformation of the first MTPJ. **(A)** Positioning the phalanx posture. **(B)** 3D-view of the first MTPJ ranging within 0-21 degrees. **(C)** 3D-view of body coordinate system. **(D)** Toggle transparency. Rotation is about axis *x*.

SF Part IX Geometric model and constraints of the first MTPJ

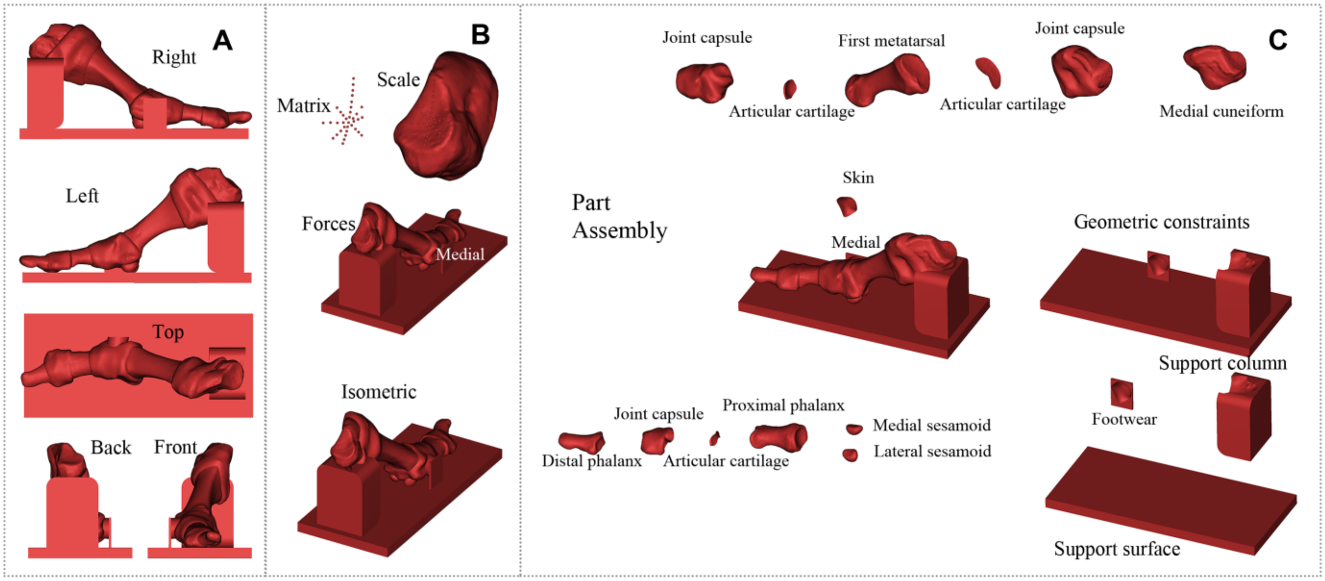

Figure S8 P1's geometric model and constraints of the first MTPJ. **(A)** Observation from different views. **(B)** Loading condition of point matrix. The direction of the force is the normal direction of the surface tangent to the action point of the force. **(C)** Parts and assembly of the first MTPJ model. (Barefooted models do not include Footwear and Skin.)

SF Part X P5's CT cross-section images before and after injury

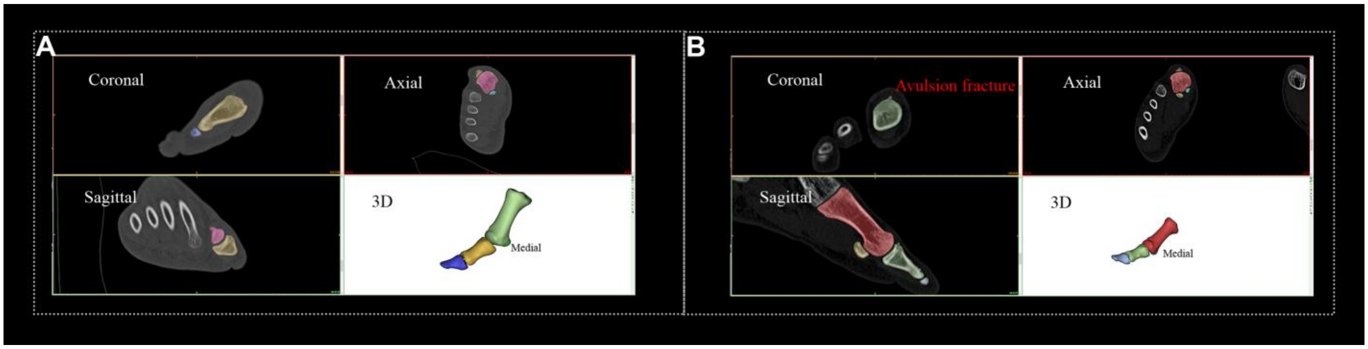

Figure S9 P5's cross-section images **(A)** before injury. **(B)** after injury. (B) shows the avulsion fracture of P5.

SF Part XI Relation between the first MTPJ angle and the stress

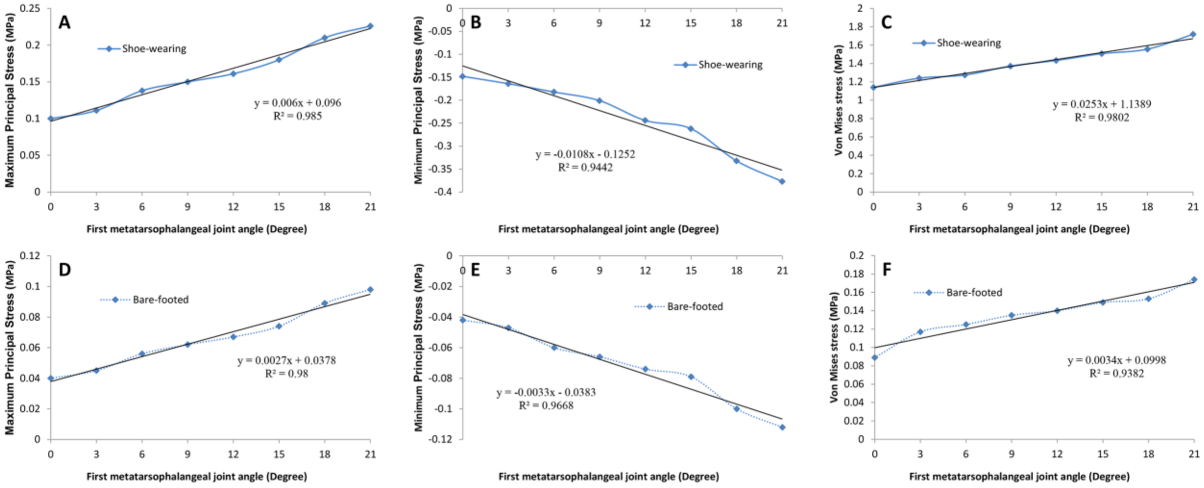

Figure S10 Relation between the first MTPJ angle and the stress. **(A)-(C)** Relation between the first MTPJ angle and the maximum, minimum principal stress and von Mises stress when wearing shoes, respectively. **(D)-(F)** Relation between the first MTPJ angle and the maximum, minimum principal stress and von Mises stress when being bare-footed, respectively. x in the equations refers the first MTPJ angle, y refers the stress, and R^2 refers to the coefficient.

SF Part XII Positions of the maximum principal stress and minimum principal stress on the joint capsule of simulated shoe wearing

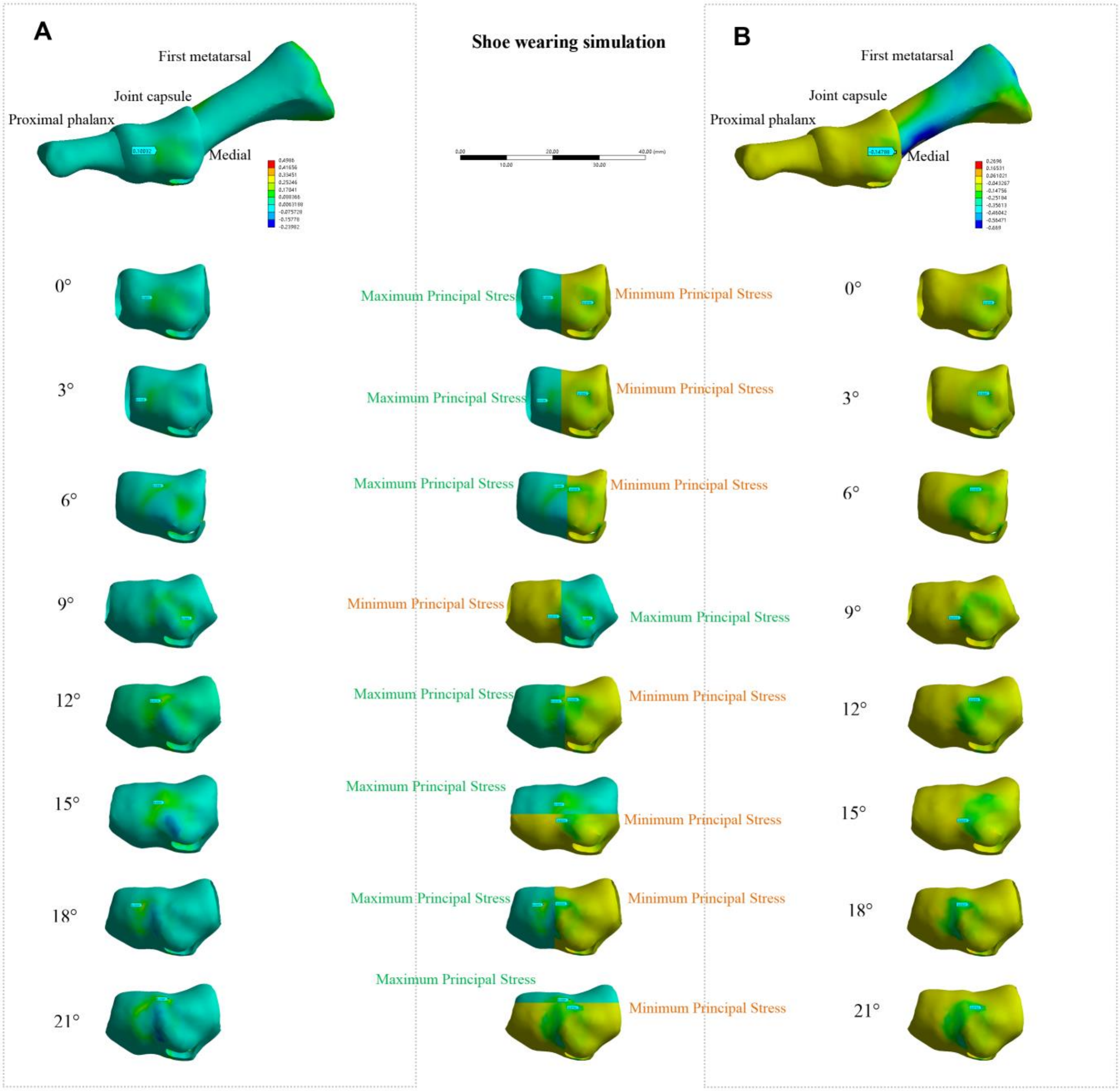

Figure S11 Maximum and minimum principal stress of shoe wearing simulation. **(A)** Maximum principal stress. **(B)** Minimum principal stress.
